# Human Milk Oligosaccharides Impact Cellular and Inflammatory Gene Expression and Immune Response

**DOI:** 10.3389/fimmu.2022.907529

**Published:** 2022-06-29

**Authors:** Fernanda Rosa, Ashok K. Sharma, Manoj Gurung, David Casero, Katelin Matazel, Lars Bode, Christy Simecka, Ahmed A. Elolimy, Patricia Tripp, Christopher Randolph, Timothy W. Hand, Keith D. Williams, Tanya LeRoith, Laxmi Yeruva

**Affiliations:** ^1^ Arkansas Children’s Nutrition Center, United States Department of Agriculture-Agricultural Research Service (USDA-ARS), Little Rock, AR, United States; ^2^ School of Veterinary Medicine, Texas Tech University, Amarillo, TX, United States; ^3^ Inflammatory Bowel and Immunobiology Research Institute, Cedars-Sinai, Los Angeles, CA, United States; ^4^ Larsson-Rosenquist Foundation Mother-Milk-Infant Center of Research Excellence, University of California San Diego, La Jolla, CA, United States; ^5^ Department of Pediatrics, University of California San Diego, La Jolla, CA, United States; ^6^ Division of Laboratory Animal Medicine, University of Arkansas for Medical Sciences, Little Rock, AR, United States; ^7^ Animal Production Department, National Research Centre, Giza, Egypt; ^8^ Center for Translational Pediatric Research, Arkansas Children’s Research Institute, Little Rock, AR, United States; ^9^ University of Pittsburgh School of Medicine, R.K. Mellon Foundation Institute for Pediatric Research, University of Pittsburgh Medical Center (UPMC) Children’s Hospital of Pittsburgh, Pittsburgh, PA, United States; ^10^ Department of Biostatistics, University of Arkansas for Medical Sciences, Little Rock, AR, United States; ^11^ Department of Biomedical Sciences & Pathobiology, Virginia Tech, Blacksburg, VA, United States

**Keywords:** human milk oligosaccharides, HMO, immunity, gastrointestinal tract, neonatal

## Abstract

Human milk harbors complex carbohydrates, including human milk oligosaccharides (HMOs), the third most abundant component after lactose and lipids. HMOs have been shown to impact intestinal microbiota, modulate the intestinal immune response, and prevent pathogenic bacterial binding by serving as decoy receptors. However, the direct effect of HMOs on intestinal function and immunity remains to be elucidated. To address this knowledge gap, 21-day-old germ-free mice (C57BI/6) were orally gavaged with 15 mg/day of pooled HMOs for 7 or 14 days and euthanized at day 28 or 35. A set of mice was maintained until day 50 to determine the persistent effects of HMOs. Control groups were maintained in the isolators for 28, 35, or 50 days of age. At the respective endpoints, intestinal tissues were subjected to histomorphometric and transcriptomic analyses, while the spleen and mesenteric lymph nodes (MLNs) were subjected to flow cytometric analysis. The small intestine (SI) crypt was reduced after HMO treatment relative to control at days 28 and 35, while the SI villus height and large intestine (LI) gland depth were decreased in the HMO-treated mice relative to the control at day 35. We report significant HMO-induced and location-specific gene expression changes in host intestinal tissues. HMO treatment significantly upregulated genes involved in extracellular matrix, protein ubiquitination, nuclear transport, and mononuclear cell differentiation. CD4+ T cells were increased in both MLNs and the spleen, while CD8+ T cells were increased in the spleen at day 50 in the HMO group in comparison to controls. In MLNs, plasma cells were increased in HMO group at days 28 and 35, while in the spleen, only at day 28 relative to controls. Macrophages/monocytes and neutrophils were lower in the spleen of the HMO group at days 28, 35, and 50, while in MLNs, only neutrophils were lower at day 50 in the 14-day HMO group. In addition, diphtheria toxoid and tetanus toxoid antibody–secreting cells were higher in HMO-supplemented group compared to controls. Our data suggest that HMOs have a direct effect on gastrointestinal tract metabolism and the immune system even in the absence of host microbiota.

## 1 Introduction

Human milk is considered the gold standard for infant’s nutrition. The World Health Organization and the American Academy of Pediatrics recommend exclusive human milk feeding of the newborn during the first 6 months of life, followed by continued human milk feeding as complementary foods are introduced for 1 year or longer (1, 2). Human milk feeding supports optimal growth, promotes gastrointestinal tract (GIT) development and immune system maturation, and protects against neonatal infections and other health outcomes beyond the neonatal phase (3–5). For instance, a lower incidence of respiratory infections has been observed in breastfed infants compared to formula-fed infants (6). Additionally, lower rates of necrotizing enterocolitis (7, 8), reduced gastrointestinal infections (9, 10), protection against wheezing and asthma (11), a reduced risk of allergy (12), and a decreased incidence of obesity in childhood (13) have been reported in breastfed infants relative to formula-fed infants. These human milk–feeding benefits are promoted by the bioactive compounds present in the human milk. Such components include milk microbiota, antibodies, immunoglobulins, hormones, lactoferrin, and human milk oligosaccharides (HMOs), among other molecules (14).

Within the human milk composition, the third most abundant solid components in human milk are HMOs after lactose and lipids. To date, more than 160 different HMO structures have been characterized. Human milk oligosaccharides are glycans naturally present in colostrum and mature milk, which are made of five basic monosaccharides: glucose (Glc), galactose (Gal), N-ethylglucosamine (GlcNAc), fucose (Fuc), and sialic acid (SA). Almost all HMOs contain lactose (Gal-B1, 4-Glc) at the reducing end (15, 16). Furthermore, it has been shown that HMOs are resistant to low pH in the stomach and pancreatic enzymes. HMOs are metabolized by the gut microbiota, and it is estimated that 1% of HMOs are likely absorbed by the intestinal epithelium and reach the systemic circulation (17–19). HMOs play an important role in protecting infants against infections and having a prebiotic effect on the gut microbiota (20–22). HMOs have been shown to stimulate gut microbiota growth and composition by serving as substrates to the LI bacteria community, specifically, *Bifidobacterium infantis*, *B. bifidum, Bacteroides fragilis*, and *B. vulgatus* (23, 24). Additionally, Roseburia and Eubacterium species that belong to *Clostridiales* metabolize HMOs as demonstrated by *in vitro* approaches (25–29). HMO–gut microbiota interactions have also been shown to promote intestinal growth by which rats supplemented with the neutral HMO 2’FL (2’-fucosyllactose) had an increase in the villus area and height (30). In addition, *in vitro* approaches and animal models have demonstrated that HMOs may exert antiviral properties by inhibiting the replication of rotavirus and by reducing diarrhea events in rats and piglets infected with rotavirus (26, 31–33). Several *in vitro* studies and mouse models also reported the immunomodulatory effects of HMOs, including the stimulation of dendritic cells (34), macrophages (35), blood mononuclear cells in piglets fed with HMOs (36), mucus production by the modulation of goblet cells (37), and enhanced humoral and cellular immune responses to influenza vaccination in mice fed with 2’FL relative to a control group (38). In contrast, most recently, HMOs have been shown to increase rotavirus infectivity (39). Enhanced cellular immune response upon dietary HMOs was also observed in rotavirus-infected neonatal piglets (40). Thus, several *in vitro* and clinical studies have demonstrated mostly the health benefits of HMOs (41, 42).

HMOs’ addition to infant formulas is an emergent field. Randomized controlled clinical trials revealed that infant formula supplemented with the HMO 2’FL was well tolerated by infants and 2’FL-formula-fed infants had similar growth compared to human milk–fed infants (43). Similarly, lower levels of pro-inflammatory cytokines were detected in the plasma of 2’FL-formula-fed infants compared to a control formula group (44). Puccio and collaborators (2017) performed a randomized controlled trial to evaluate the effects of a dairy-based milk formula supplemented with HMOs 2’FL + LNnT (lacto-N-neotetraose). Lower respiratory tract infections were reported for the infants receiving the 2’FL + LNnT-formula compared to the control-formula-fed infants through 12 months of age (45). Furthermore, a mixture of 5 HMOs (2’-FL, 3-FL, LNT, 3’-SL, and 6’-SL) at 5.75 g/L supplemented in infant formula was well tolerated and improved overall growth in healthy infants during the first 4 months of life (46). Although these are promising findings as alternatives to human milk feeding, future research to evaluate the immunological effects of an individual or combined HMO mixture is needed to demonstrate the benefits to infant health.

It is clear that HMOs provide different health benefits through gut microbiota modulation during infancy. Despite all *in vitro* and clinical studies, the immediate effect of HMOs in the short term and any persistent effect beyond weaning remains to be elucidated. Moreover, determining the direct effect of HMOs in the absence of microbiota is an underappreciated area of research. For instance, the binding and absorption specificity in the intestinal tissues by HMOs without bacteria interaction, as well as the direct immune-modulatory effects of HMOs on the host, remain to be evaluated. Therefore, to address the gap in knowledge, this study investigated the direct effect of HMOs on the GIT and immune system using a preclinical germ-free (GF) mouse model.

## 2 Materials and Methods

### 2.1 Germ-Free Mice Experiments

All mouse experiments were conducted in accordance with the Institutional Animal Care and Use Committee at the University of Arkansas for Medical Sciences. Weaned C57BL/6J male and female mice at 21 days of age were housed in plastic flexible film isolators at 23°C under a strict 12-h light cycle (lights on at 0700h and off at 1900h). Animals had *ad libitum* access to sterilized food and water. At 21 days of age, mice (n = 7–18 per group; with a specific ratio of females vs. males are mentioned in the results of each figure and table) were weighed and distributed across groups to balance the initial body weights. HMOs were isolated and purified from the donor human milk and were obtained from LB’s Laboratory at University of California (UC)-San Diego (47). Mice were orally gavaged with 15 mg/day of pooled HMOs (48) based on a previous study from LB’s team that showed a reduction in the colonization of *Escherichia coli* attachment in suckling mice. To determine the immediate effects, mice received HMOs either for 7 or 14 consecutive days and were euthanized at day 28 or 35, respectively. To determine the persistent effects post- weaning of HMO feeding, another set of mice was orally gavaged with 15 mg/day of pooled HMOs for 7 or 14 consecutive days and maintained in the isolator until euthanasia on day 50. To determine the direct effect of HMOs on vaccine response, a subset of mice euthanized at day 50 was also immunized on days 21 and 35 with 10 µg/mouse of cholera toxin through oral gavage and intraperitoneal injection of 100 µl/mouse of Pediarix (immunization against diphtheria, tetanus, and pertussis). Control groups were maintained in the isolators for 28, 35, or 50 days of age.

### 2.2 Sample Collection and Measurements

At the respective endpoints (i.e., day 28, 35, or 50), mice were euthanized with isoflurane (1%–5%) in a chamber. After breath cessation, mice were subjected to cervical dislocation as secondary means to ensure death. SI and LI lengths were measured. Tissue samples were processed fresh or fixed in formalin or flash frozen in liquid nitrogen and stored at -80°C for further flow cytometry, histomorphometric, and transcriptomic analyses, respectively.

### 2.3 Histomorphometric Analyses

The distal ileum (3 cm), proximal colon (2 cm), and cecal sections were fixed in formalin and the histomorphometric analyses of small intestinal (SI) crypt depth and villi height, and the LI and cecum gland depth were carried out to evaluate the GIT morphology. Formalin-fixed tissues were cut, paraffin-embedded, and processed for staining with hematoxylin and eosin (H and E). All measurements were made using Aperio Image software by a board-certified pathologist TL. Crypts immediately adjacent or as near as possible to measured villi were selected and the crypt depth was measured using the pen tool. Crypts had to be intact and entirely within the plane of section. Intestinal length data at 28 and 35 days of age were analyzed using the GLM procedure of SAS 9.4 (SAS Institute, Inc., Cary, NC, USA), while the data at 50 days of age were analyzed using the PROC MIXED procedure of SAS. Intestinal histomorphometric data were analyzed using R. Group × sex interactions were assessed by the Analysis of Deviance Table (Type II Wald chi-square test) at days 28 and 35. At day 50, group × sex interactions were assessed by permutational multivariate ANOVA (PERMANOVA). Statistical significance was declared at *P-*adjusted ≤ 0.05.

### 2.4 RNA Isolation, Library Preparation, and Sequencing

At the respective endpoints, small intestinal (SI) and large intestinal (LI) tissues were subjected to transcriptome analysis. Total RNA was extracted using TRIzol reagent, and the RNA was purified using a miRNeasy kit from Qiagen following the manufacturer’s standard instructions. The RNA concentration was measured by Nanodrop with the average ng/µl of 1,268.96 ± 557.47 and 768.36 ± 502.19 (mean ± SD) for SI and LI samples, respectively. The RNA quality was measured using the high-sensitivity RNA ScreenTape^®^ at TapeStation analysis software A.02.02-SR1 (Agilent Technologies, Inc. 2017). The average RNA integrity number (RIN) for the SI and LI samples was 5.05 and 7.0, respectively. Sequence-ready libraries were prepared from total RNA using the Illumina TruSeq stranded messenger RNA (mRNA) Sample Prep kit and following the manufacturer’s protocol. Briefly, mRNA is selected from total RNA and subsequently converted to complimentary DNA (cDNA). 3’ends are adenylated and then ligated with a unique index adapter followed by PCR enrichment. Quality control was performed using an Agilent Fragment Analyzer and Thermofisher Qubit fluorometer. Libraries were normalized and pooled, then sequenced using an Illumina NextSeq 500 series sequencer. All sequencing procedures were conducted by the Center for Translational Pediatric Research Genomics Core Lab at Arkansas Children’s Research Institute (Little Rock, AR, USA).

### 2.5 RNA-Seq Alignment, Differential Expression, and Pathway Analysis

Sequence reads were aligned to a genome index that includes both genome sequence (GRCm38 mouse primary assembly) and the exon/intron structure of known mouse gene models (Gencode M25 comprehensive genome annotation) using STAR ultrafast universal RNA-seq aligner v2.7.8a (49). Alignment files were used to generate strand-specific gene counts. Independent filtering was applied to remove low-count genes, and only protein-coding genes were considered for downstream analysis. This masked set included a total of 16,506 genes. Expression estimates provided throughout were computed in the units of transcripts per million (TPM). Normalized count and variance-stabilized data were used for all ordination, differential, and clustering analyses. The Gene Expression Deconvolution Interactive Tool (GEDIT) was used to quantify the cell type from gene expression data (50). Gene signatures from the *Tabula Muris* single-cell reference database were used to estimate cell type proportions from TPM data (51). Among all signatures, the estimated proportion of epithelial cells was the highest for most samples but with some variability and was hence incorporated as an additional term in our multivariate models to correct for the influence of tissue heterogeneity on overall expression estimates.

Principal component analysis was performed with the *prcomp* function in R using variance-stabilized data. Differential expression analyses were performed in DESeq2 Bioconductor package in R (52). In exploratory analyses, we performed pair-wise comparisons between two groups of samples and identified significantly discriminating genes based on Benjamini–Hochberg adjusted Wald test *P*-value > 0.05. Variance partition analysis was performed to quantify the contribution of each variable to overall variation in gene expression using the *variancePartition* function in R (53). The global linear mixed model included terms for age (endpoints), treatment (dietary intervention: control, 7-day or 14- day HMO feeding), tissue type (SI or LI), sex, cage, immunization, and estimated epithelial proportion. As anticipated, tissue type showed maximum variance for most genes. Tissue-specific models discussed throughout included all other terms and were used to identify genes most associated with age and treatment after correcting for all other factors. Specifically, genes that showed maximum variance in response to treatment and age (endpoints: 21, 28, 35, and 50 days) were selected for downstream analysis. In total, 2,526 and 2,731 genes in SI and LI, respectively, were considered for model-based co-expression analysis using MBCluster.Seq (54). We employed this unsupervised approach to classify genes in 20 different clusters for SI and LI. The expression of these modules was reported across different time points and treatments. Functional and pathway enrichment analyses for the genes in each cluster were carried out using Metascape (55).

### 2.6 Flow Cytometry

Mesenteric lymph nodes (MLNs) and spleen were collected from each mouse in a collection tube containing cold Roswell Park Memorial Institute media (RPMI) with 20% FBS media and immediately stored on ice. Cells were isolated using the protocol previously described (46). Single-cell suspensions were counted using Trypan Blue and stained with live/dead stain followed by the Fc receptor block (Innovex, NB309). Stained cells were analyzed using BD LSRFortessa (UAMS flow cytometry core facility). Antibody information is provided in **Supplementary Table 1**. Gating for T cells, B cells, and myeloid cells are shown in **Supplementary Figure 1**. Statistical significance was determined using the Mann–Whitney test in GraphPad Prism Version 9.3.1 (www.graphpad.com), and p<0.05 was considered significant.

### 2.7 ELISA Assay

CTB-, TT- and DT-specific antibody titers were measured using enzyme-linked immunosorbent assay (ELISA) as described previously (56). Flat microtiter plates were coated with CTB (1 µg/ml) + 0.3 µM monosialoganglioside diluted in 0.5 M NaHCO_3_, TT (5 µg/ml), or DT (5 µg/ml) and incubated at 4°C for 24 h. All wells were blocked with a 10% BSA blocking buffer and incubated at 37°C for 1 h followed by the serial dilution of 5 µl of the serum sample + 195 µl of the 10% BSA blocking buffer into sample wells and incubation for an hour at 37°C. Plates were washed 3 times with the 5% BSA wash buffer and 100 µl of diluted HRP-conjugated goat anti-mouse IgG (1:20,000 dilution, Southern Biotech, 1040-05) or goat anti-mouse IgA (1:20,000 dilution Southern Biotech, 1040-05) antibodies were added into respective wells and incubated at 37°C for 1 h. After incubation, plates were washed 3 times with the 5% BSA wash buffer and 100 µl of the TMB substrate (Thermofisher, 34022) was added into each well and incubated at room temperature in the dark for 30 min. 100 µl of 2M stop solution (sulfuric acid) was added into each well to stop the reaction and absorbance was measured using Omega Polar Star Absorbance Reader at 450 nm. Absorbance from the last dilution that resulted in twice the signal of the background was converted to log 10. Statistical significance between control and treatment groups were determined using one-way ANOVA with Bonferroni’s multiple comparison correction in GraphPad Prism Version 9.3.1, and the adjusted p-value <0.05 was considered significant.

### 2.8 ELISpot Assay

In house developed ELISpot assay was used to assess the secretion of antibodies for Cholera toxin subunit B (CTB)-IgA, Tetanus toxoid (TT)-IgG and Diphtheria toxoid (DT)-IgG from MLN and spleen as described previously (56). ELISpot plates (Millipore, MSIPS4W10) were coated with 20 µg/ml of CTB + 3 µM monosialoganglioside (Millopore Sigma, G7641) or 25 µg/ml of TT and 3.95 µg/ml of DT and incubated at 4°C overnight in dark. After incubation, plates were washed 5 times and the membrane was blocked by adding RPMI + 10% FBS and incubated for 1 h at room temperature. Cells (2 × 10^5^ to 2 × 10^6^ cells for spleen and 1.25 × 10^5^ to 1 × 10^6^ for MLN) were plated in complete media (RPMI, 10% heat-inactivated FBS, 1 mM sodium pyruvate, 1 mM NEAA, 1 mM HEPES, fungizone, 0.1% vancomycin of 50 mg/ml, 1% penicillin/streptomycin, 2-ME), and incubated at 37°C, in 5% CO_2_ for 72 h. Plates were washed 5 times with 1× PBS and 50 µl/well of goat anti-mouse IgA (1:1,000 dilution, Novus Biologicals, NB7502 Englewood, CO) (into CTB wells) and goat anti-mouse IgG (1:1,000 dilution, Bio-Rad, 103004) (into TT and DT wells) antibodies diluted in 1% BSA, conjugated to alkaline phosphatase were added and incubated for 1.5 h at 37°C. Plates were washed 5 times with 1× PBS and 100 µl of filtered BCIP/NBT-plus (Mabtech, 3650-10) added into each well and incubated at room temperature for 30 min. Plates were washed twice with deionized water to stop the color development and dried. Antibody-secreting cells (ASCs) were counted using Nikon 7645 microscope or Nikon NI-150 Fiber Optic Illuminator and normalized per 1 × 10^6^ cells. Statistical significance between control and treatment groups were determined using one-way ANOVA with Bonferroni’s multiple comparisons correction in GraphPad Prism Version 9.3.1 and the adjusted p-value <0.05 was considered significant.

### 2.9 Lymphocyte Proliferation Assay

Cell-mediated immunity from MLN and the spleen was examined by measuring proliferation in response to CTB, TT, or DT as described previously (56). Isolated MLN or spleen cells were maintained in complete media. Approximately 5 × 10^4^ to 1.5 × 10^5^ (MLN) or 1 × 10^5^ to 3 × 10^5^ (spleen) cells were added in duplicates into a 96-well cell culture plate and stimulated by adding 10 µg/ml of CTB or 3.3 µg/ml of TT or 1.2 µg/ml of DT. For each sample, the duplicates of equal number of cells were used as control. Plates were incubated at 37°C, 5% CO_2_ for 72 h. AlamarBlue (20 µl, DAL1100; Thermo Fisher Scientific Waltham, Massachusetts) was added into each well and incubated for 24 h, and fluorescence was measured on Omega Polar Star Absorbance Reader. The statistical significance between control and treatment groups were determined using one-way ANOVA with Bonferroni’s multiple comparisons correction in GraphPad Prism Version 9.3.1, and the adjusted p-value <0.05 was considered significant.

## 3 Results

### 3.1 Gastrointestinal Tract Development

#### 3.1.1 Intestinal Length

The SI length did not differ between control and HMO groups at 28 or 35 days of age (**Table 1**). However, the LI was longer in the mice that received HMOs for 7 days compared to the control mice at 28 days of age (*P* = 0.05; **Table 1**). At 50 days of age, the length of the SI also did not differ between control and treated mice (**Table 2**). The LI was longer in the mice that received HMO for 7 or 14 days relative to the control group (*P* < 0.01; **Table 2**).

**Table 1:** Intestinal length for germ-free mice used as control compared to the HMO groups at 28 or 35 days of age.

	Group^1^				
Length (cm)	Control	SD	HMO 7 d	SD	*P* ^2^
Small intestine	28.15	6.78	29.01	1.88	0.74
Large intestine	4.20	1.03	5.17	0.77	0.05
Length (cm)	**Control**	SD	**HMO 14 d**	SD	*P* ^2^
Small intestine	31.30	3.33	32.38	2.39	0.47
Large intestine	4.94	1.30	5.80	0.72	0.16

For each group, the average length is presented along with the standard deviation per group (SD).

^1^Groups: Control = germ-free mice euthanized either at 28 days of age (n=18; M = 11, F = 7) or 35 days of age (n=10; M = 5, F = 5); HMO 7 d = germ-free mice that received 100 µl of HMO (15 mg/day) through 7 consecutive days and euthanized at 28 days of age (n=7; M = 3, F = 4); HMO 14 d = germ-free mice that received 100 µl of HMO (15 mg/day) through 14 consecutive days and euthanized at 35 days of age (n=7; M = 3, F = 4). ^2^P-values for group comparisons are shown.

**Table 2:** Intestinal length for germ-free mice used as control compared to the HMO groups at 50 days of age.

	Group^1^						
Length (cm)	Control	SEM	HMO 7 d	SEM	HMO 14 d	SEM	*P* ^2^
Small intestine	30.60	1.6	28.50	1.84	32.68	2.30	0.36
Large intestine	5.15^a^	0.30	6.85^b^	0.35	6.80^b^	0.43	< 0.01

For each group, the average length is presented along with the standard error of the mean (SEM).

^1^Groups: Control = germ-free mice euthanized at 50 days of age (n=5; M = 3, F = 2); HMO 7 d = germ-free mice that received 100 µl of HMO (15 mg/day) through 7 consecutive days and euthanized at 50 days of age (n=18; M = 11, F = 7); HMO 14 d = germ-free mice that received 100 µl of HMO (15 mg/day) through 14 consecutive days and euthanized at 50 days of age (n=11; M = 8, F = 3). ^2^The P-value for group comparisons are shown. ^a,b^Different superscripts represent the significance difference between groups.

#### 3.1.2 Intestine Morphology

**Figure 1:**
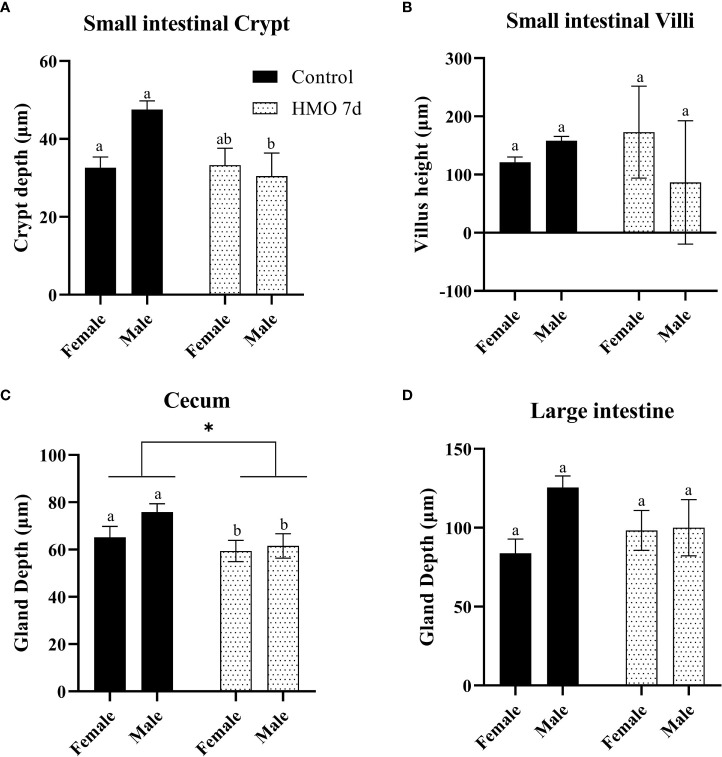
Histomorphometric parameters analyzed in the control groups (n=18; M = 11, F = 7) and in the mice that received human milk oligosaccharides (HMOs) for 7 days and euthanized at 28 days of age (n=7; M = 3, F = 4). **(A)** Small intestinal (SI) crypt depth (µm). **(B)** SI villus height (µm). **(C)** Cecal gland depth (µm). **(D)** Large intestinal gland depth (µm). ^a,b^Different superscripts represent the statistical difference between individual groups. *Represents the statistical difference between groups regardless of sex.

**Figure 2:**
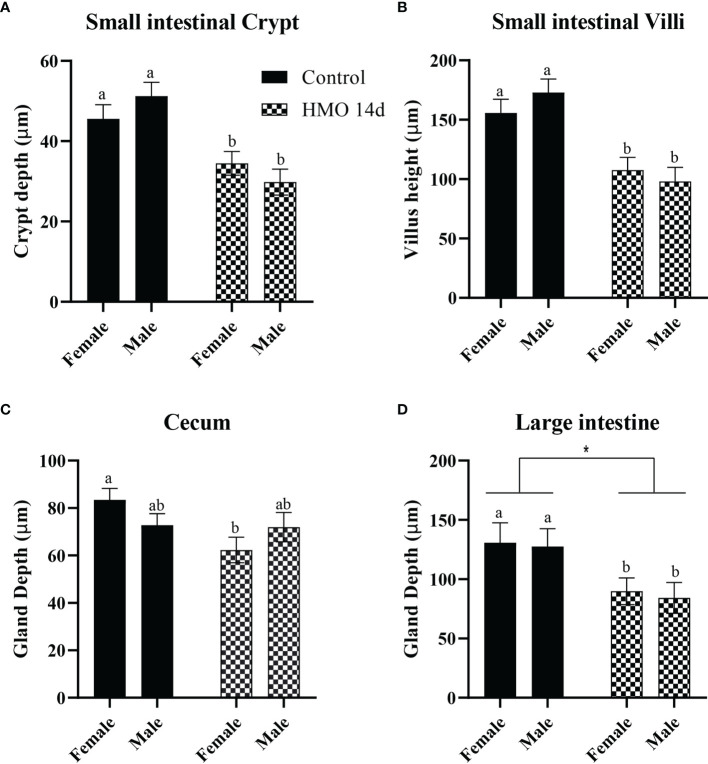
Histomorphometric parameters analyzed in the control groups (n=10; M = 5, F = 5) and in the mice that received HMOs for 14 days and euthanized at 35 days of age (n=7; M = 3, F = 4). **(A)** SI crypt depth (µm). **(B)** SI villus height (µm). **(C)** Cecal gland depth (µm). **(D)** Large intestinal gland depth (µm). ^a,b^Different superscripts represent the statistical difference between individual groups. *Represents the statistical difference between groups regardless of sex.

The statistical significance for the group (control vs. HMO 7d vs. HMO 14d), sex, and their interactions for SI villi and crypt and LI and cecal gland depth at 28 and 35 days of age are shown in **Supplementary Table 2**.A group × sex interaction was observed for the SI crypt depth (P < 0.01). No group × sex interaction was observed for the SI villi height (*P* = 0.10) and LI or cecal depth (*P* ≥ 0.06). At 28 days of age, a group × sex interaction for the SI crypt was reflected by a decrease in the crypt depth (*P* = 0.01) of the HMO 7 d males compared to the control males (**Figure 1A**). The villus height was not statistically different between groups or by sex (**Figure 1B**). The cecal gland depth decreased in the mice that received HMO for 7 d compared to the control mice, regardless of sex (*P* = 0.05; **Figure 1C**). The LI gland depth was not affected by treatment or sex (**Figure 1D**). At 35 days of age, the SI crypt depth was decreased in the HMO 14 d group compared to the control group for both males and females (*P* ≤ 0.02; **Figure 2A**). Meanwhile, for the SI villi heights, a group (*P* < 0.001) and a sex (*P* = 0.02) effect was observed. The villi heights were decreased in the HMO 14 d group relative to the control (**Figure 2B**). A group effect was observed for the cecum, which was reflected by a decrease (*P* = 0.02) in the gland depth of the females-HMO 14 d relative to the females-control (**Figure 2C**). Regardless of sex, the LI gland depth decreased in the HMO 14 d group compared to the control group (*P* = 0.002; **Figure 2D**).

**Figure 3:**
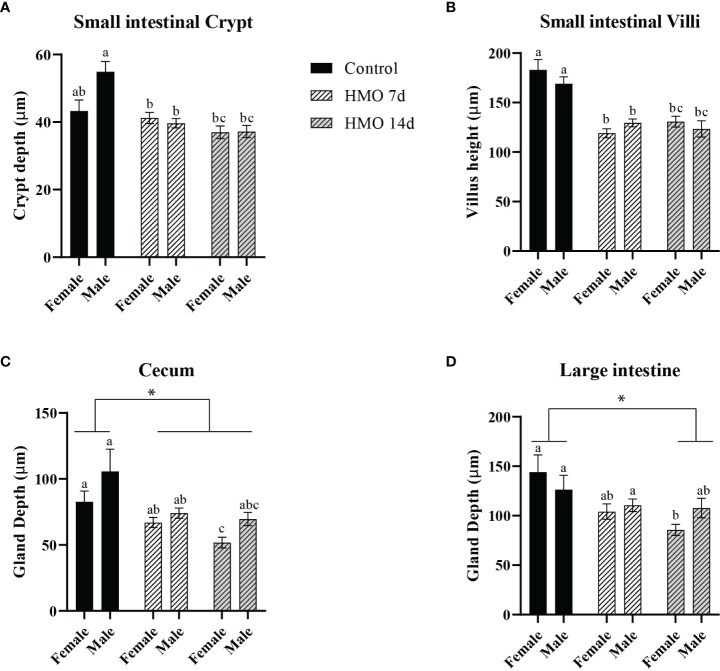
Histomorphometric parameters analyzed in the control groups (n=5; M = 3, F = 2) and in the mice that received HMOs either for 7 days (n=18; M = 11, F = 7) or for 14 days (n=11; M = 8, F = 3) and euthanized at 50 days of age, respectively. **(A)** SI crypt depth (µm). **(B)** SI villus height (µm). **(C)** Cecal gland depth (µm). **(D)** Large intestinal gland depth (µm). ^a,b,c^Different superscripts represent the statistical difference between individual groups. *Represents the statistical difference between groups regardless of sex.

The PERMANOVA statistical analysis for the HMO groups, sex, and their interactions at 50 days of age are shown in **Supplementary Table 3**. A group × sex interaction (*P* = 0.03) was observed for the SI crypt, which was reflected by a decrease in the crypt depth in the male mice of both HMO groups compared to the male mice in the control group (*P* < 0.01; **Figure 3A**). The villus height of the SI of mice treated with HMO for 7 or 14 days was decreased (*P* < 0.01) compared to the control mice at 50 days of age. However, there was no difference in the SI crypt depth and villus height between the two HMO groups (**Figures 3A, B**). The female mice that received HMO for 14 days had a decreased cecal gland depth compared to the female mice of HMO 7 d and the female mice in the control group (*P* ≤ 0.02; **Figure 3C**). Overall, the mice that received HMO for 7 or 14 d independent of sex had a decrease in the cecal depth compared to the control group at 50 days of age (*P ≤ 0.03; **Figure 3C**). The LI gland depth was decreased in the female mice of the HMO 14d relative to the females in the control group (*P* = 0.01; **Figure 3D**). Regardless of sex, the LI depth of the HMO 14 d group was decreased compared to the control group at 50 days of age (*P = 0.02; **Figure 3D**).

### 3.2 Differential Gene Expression

#### 3.2.1 HMO Impact on Gene Expression

**Figure 4:**
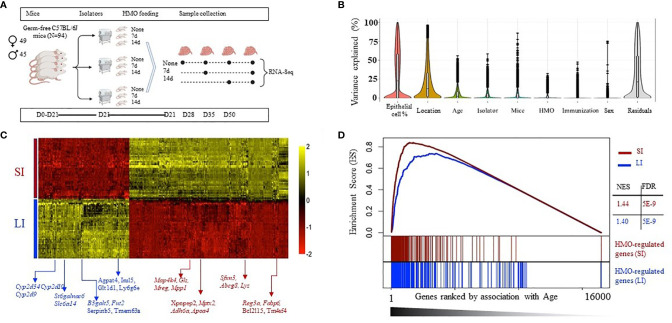
**(A)** Experimental schema with mice weaned at 21 days of age (n=3 F) and HMO feeding for 7 and 14 days in mice sacrificed at 28, 35, and 50 days. Samples collected from SI and large intestine (LI) at each time point were subjected to RNA-seq to determine the effect of HMO feeding and age on overall gene expression patterns. **(B)** Variance partition analysis using a global model to find out the contribution of each variable to the overall gene expression variance. **(C)** Heat map of the top 300 genes most associated with the tissue type in multivariate models, showing maximal location-specific expression; **(D)** Gene enrichment analysis (GSEA) to estimate the degree of overlap between age-induced and HMO-induced expression alterations. Separately in LI and SI samples, genes were ranked based on variance explained due to age (x-axis), and the segment plots (bottom) highlight the position of the top 300 genes most affected by HMO. The vertical axis in line plots (top) represents the cumulative enrichment score (ES) from GSEA analysis, and NES is the overall normalized enrichment score (with FDR = false discovery rate). Groups (conditions): Control germ-free mice (GF) euthanized either at 28 days of age (n= 9; M=5, F=4) or 35 days of age (n= 5; M=3, F=2) or 50 days of age (n = 5; M=1, F=4) respectively; 7d HMOs euthanized at d28 = GF mice that received 100 μl of HMO through 7 consecutive days and euthanized at 28 days of age (n= 5; M=2, F=3); HMO_14d_D35 = GF mice that received 100 μl of HMO through 14 days and euthanized at 35 days of age (n= 6; M=3, F=3); HMO_7d_D50 = GF mice that received 100 ul of HMO through 7 consecutive days and euthanized at 50 days of age (n= 5; M=1, F=4); HMO_14d_D50 = GF mice that received 100 μl of HMO through 14 consecutive days and euthanized at 50 days of age (n= 10; M=7, F=3).

A highly replicated combinatorial transcriptomic design (**Figure 4A**) was used to delineate gene expression changes in the neonatal GF mice with 7 or 14 days of HMO supplementation. To control for unwanted sources of variation, we first applied gene expression deconvolution using mouse single-cell expression signatures. This analysis revealed maximal enrichment in epithelial gene expression for most samples (**Supplementary Dataset S1**), dampening the effect of tissue heterogeneity on downstream analysis. In addition, the expression dataset was analyzed using mixed-effects multivariate models and variance-partition analysis to estimate the separate contribution to gene expression variability of all experimental and technical factors in the experiment (**Figure 4B**). Expectedly, we first observed that regional variability between SI and LI samples was dominant for many genes (**Figure 4B** and **Supplementary Dataset S2**). The location-specific expression of known SI and LI marker genes was recapitulated as shown in **Figure 4C**, which included a differential activity in transport, absorption, and secretion pathways (**Supplementary Dataset S3**), the restricted expression of Paneth cells (i.e., defensins, *Lyz*, *Mptx2*), and SI enterocyte genes (*Adh6a*, *Apoa4*, *Reg3a*, *Fabp6*) in SI samples. Increased expression of the mucus layer and colon-specific cytochrome P450 enzymes (*St6galnac6*, *B3galt5*, *Fut2*, *Cyp2d34*, *Cyp2d10*, *Cyp2d9*) among others, was detected in LI samples. In addition, we observed that both time of sampling (age) and HMO feeding had significant contributions to gene expression variability (**Figure 4B** and **Supplementary Dataset S2**). Strikingly, gene set enrichment analysis (GSEA) showed that the genes most affected by HMO were also significantly associated with age in both SI and LI (**Figure 4D**), suggesting that HMO feeding interacts with pathways involved in tissue maturation. Therefore, all subsequent analyses were performed, accounting for the interaction between both factors.

#### 3.2.2 Cluster of Genes Impacted by HMOs

**Figure 5:**
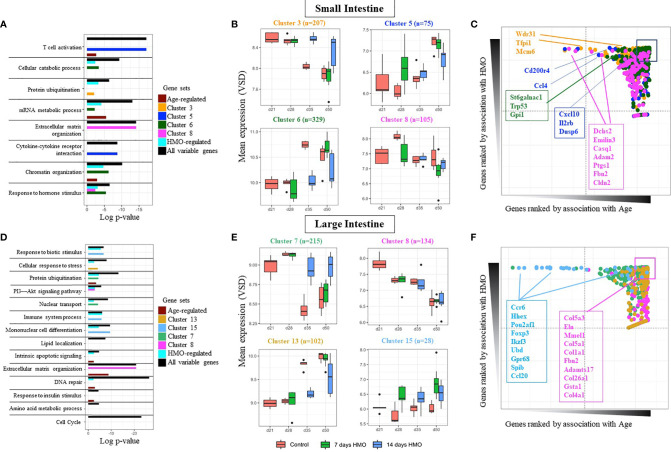
Gene cluster–based analysis for SI samples **(A)** Pathway enrichment based for several gene classes: *All variable genes* (highly variable genes regardless of factor); *Age* (genes most affected by age), *Condition* (genes most affected by HMO feeding), and for specific clusters of co-expressed genes (shown here for clusters 3, 5, 6, and 8). **(B)** Gene expression profile of selected gene clusters in SI samples. Each bar represents data from at least 5 replicates. **(C)** Rank distribution plot of selected genes in each SI cluster in **(B)** highlighting individual contributions of age and HMO to the gene’s expression profile. **(D–F)** same as above for large intestine samples (LI). GF mice weaned at 21 days of age (n=3 F); Groups (conditions): Control GF mice euthanized either at 28 days of age (n= 9; M=5, F=4) or 35 days of age (n= 5; M=3, F=2) or 50 days of age (n= 5; M=1, F=4) respectively; 7d HMOs euthanized at d28 = GF mice that received 100 ul of HMO through 7 consecutive days and euthanized at 28 days of age (n= 5; M=2, F=3); HMO_14d_D35 = GF mice that received 100 μl of HMO through 14 days and euthanized at 35 days of age (n= 6; M=3, F=3); HMO_7d_D50 = GF mice that received 100 μl of HMO through 7 consecutive days and euthanized at 50 days of age (n= 5; M=1, F=4); HMO_14d_D50 = GF mice that received 100 μl of HMO through 14 consecutive days and euthanized at 50 days of age (n= 10; M=7, F=3).

Due to the differences in baseline expression and the distinct functionality of the gut epithelium from both locations, separate downstream analyses for SI and LI samples were performed. In each location, we again confirmed a significant contribution of age and HMO to gene expression variance for many genes in both SI and LI (**Supplementary Dataset S4**, **Supplementary Figure 2**). Both factors were found to have a significant interaction (**Figure 4D**). Interestingly, the pool of all variable genes associated with age, HMO feeding, or their combination was enriched by homeostatic and cellular metabolism ontologies (i.e., *extracellular matrix, mRNA processing, chromatin organization, and response to hormone stimulus*) and interestingly with immune response and activation, regardless of the location (black bars in **Figure 5A** and **Supplementary Dataset S5**, **S6**). However, we aimed to characterize in detail HMO-specific changes. To this end, the expression of all variable genes was analyzed using unsupervised model-based clustering techniques to identify clusters of genes sharing a distinct expression trend (**Supplementary Figure 3** and **Supplemental Figure 4**). For instance, in the SI, we observed several gene clusters with age-related changes in gene expression strongly modulated by HMO feeding (**Figure 5B** and **Supplementary Figure 3**). In fact, multivariate analyses showed that a fraction of these genes were specifically associated with HMO treatment but not with age (high HMO rank, low Age rank - **Figure 5C**). The clusters of co-expressed genes showed significant associations with specific functional processes (**Figure 5A** and **Supplementary Dataset S5**). For instance, genes showing gradual increased expression with time were significantly associated with immune responses and cytokine signaling (i.e., clusters 4, 5, and 15, **Figure 5B** and **Supplementary Figure 3**), which showed lower expression levels for mice in the 14-day HMO-feeding group at day 50. In contrast, gene cluster 6 (**Figure 5B**) showed greater expression of genes involved in hormone stimulus and chromatin organization in the SI of the 7-day HMO-feeding group at day 50. Similarly, this unsupervised analysis revealed the clusters of extracellular matrix genes with decreased temporal expression (cluster 8) in some cases with apparent HMO modulation and associated with protein ubiquitination and carbohydrate metabolism (e.g., cluster 3).

Similar analyses on genes significantly regulated in the LI revealed the enrichment in key signaling pathways, including the cell cycle, extracellular matrix, and several metabolic processes (**Figure 5D**). The full list of genes and clusters impacted by HMO feeding and/or age is presented in the **Supplementary Dataset S6** and **Supplementary Figure 4**, respectively. Briefly, genes significantly affected by HMO feeding in the LI were often annotated in the regulation of immune system processes and the response to biotic stimuli, among others (**Figure 5D** and **Supplementary Dataset S6**). According to the clustering sets (**Figures 5E, F**), at 35 and 50 days of age, several genes involved in the protein ubiquitination within cluster 7 (i.e., *Ube2v2, Ube3a, Ubqln2, Ltn1, and Tgfbr1*, among others; **Supplementary Dataset S6**), as well as the nuclear transport pathway within cluster 7 (i.e., *Alkbh5, Eny2, Xpo4, and Tnpo*, among others; **Supplementary Dataset S6**) had higher expression in the LI of the 14-day HMO-feeding group compared to the control and 7-day HMO-feeding groups. At day 50, HMO treatment increased the expression of genes (i.e., *CCR6*, *HHEX*, *POU2AF1*, *CCL20*, *FOXP3*, *IKZF3*, *UBD*, *GPR68*, *SPIB*, and *IFI206*; **Supplementary Dataset S6**) involved in the mononuclear cell differentiation within cluster 15 (**Figures 5D, E**) relative to the control group. Furthermore, decreased expression with age without apparent modulation by HMO feeding was observed within cluster 8 (**Figures 5D, E**). Such enrichment of age-associated genes was maximal for extracellular matrix organization (i. e., *COL3A1*, *COL4A1*, *COL4A5*, *COL5A1*, *COL1A1*, and *COL1A*, among others; **Supplementary Dataset S6**).

### 3.3 Immune Cell Composition

**Figure 6:**
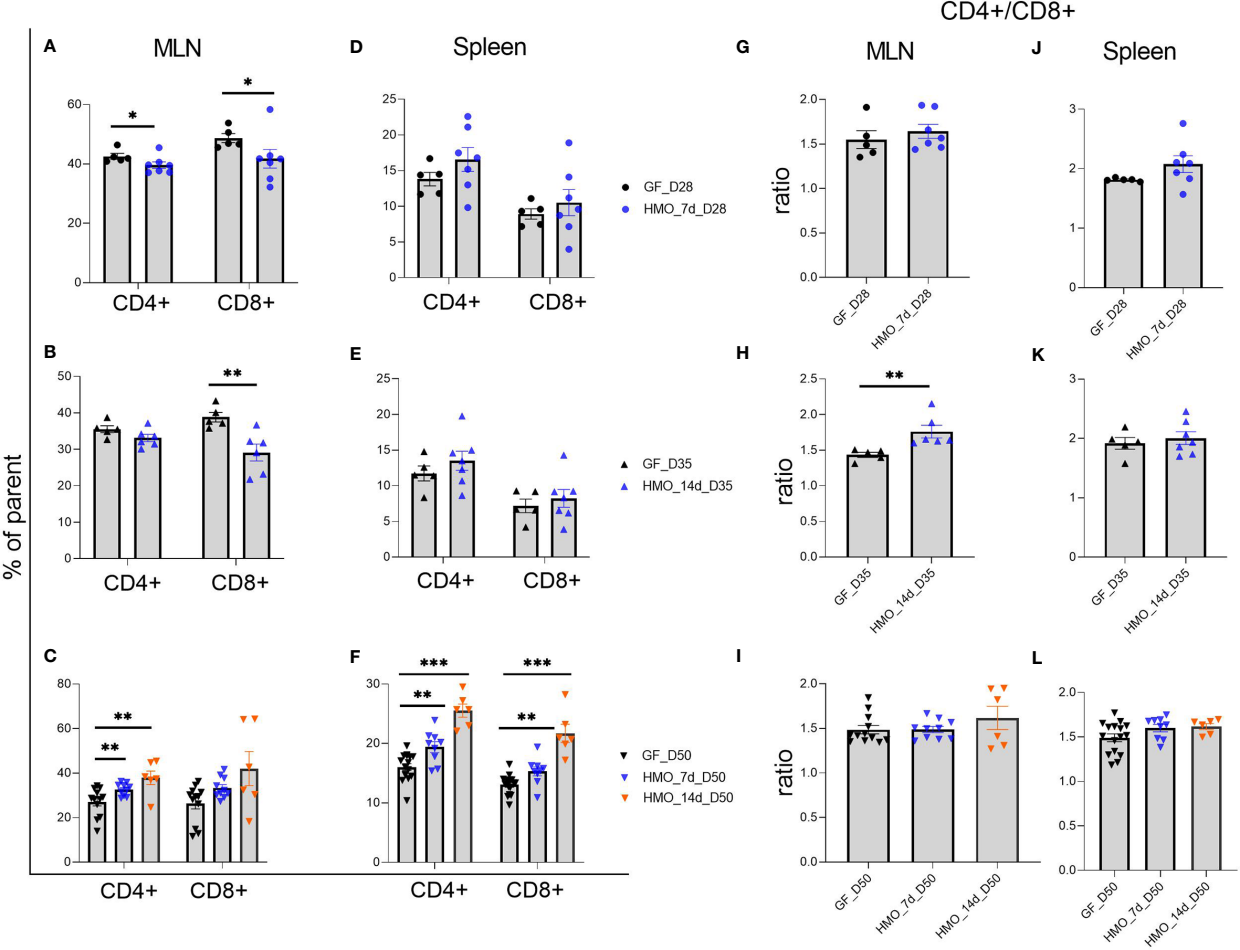
T cells (CD3+ CD4+ and CD3+ CD4- CD8+) composition in MLN and spleen following HMO administration. **(A–C)** T-cell composition in MLN. **(D–F)** T-cell composition in spleen. Cell populations are shown as the percentage of parent population (**Supplementary Figure 1A**). **(G–I)** Ratio of CD4+ T cells to CD8+ T cells in MLN. **(J-L)** Ratio of CD4+ T cells to CD8+ T cells in spleen. Ratio was calculated using absolute numbers of CD4+ and CD8+ cells. Each dot represents a mouse, and data are shown on mean ± SEM. Statistical analysis was performed using the Mann–Whitney test in GraphPad Prism Version 9.3.1 (www.graphpad.com). Statistical significance: *** p<0.001, **p<0.01, *p<0.05, ns, not significant. Groups: Control (GF_D28, GF_D35, and GF_D50) = germ-free mice (GF) euthanized either at 28 days of age (n= 5; M=2, F=3) or 35 days of age (n= 5; M=2, F=3) or 50 days of age (n= 16; M=8, F=8) respectively; HMO_7d_D28 (n= 7; M=3, F=4) = GF mice that received 100 μl of HMO through 7 consecutive days and euthanized at 28 days of age; HMO_14d_D35 (n= 7; M=3, F=4) = GF mice that received 100 μl of HMO through 14 days and euthanized at 35 days of age; HMO_7d_D50 (n= 11; M=6, F=5) = GF mice that received 100 μl of HMO through 7 consecutive days and euthanized at 50 days of age; HMO_14d_D50 (n= 6; M=5, F=1) = GF mice that received 100 μl of HMO through 14 consecutive days and euthanized at 50 days of age.

Immune cell composition data are shown in **Figures 6** **–** **8**. In MLN, 7 and 14 days of HMO supplementation decreased CD4+ and CD8+ T cells at 28 and 35 days of age, respectively (**Figures 6A, B**) in comparison to GF controls. Interestingly, the CD4+ T-cell population increased at day 50 of 7- and 14-day HMO groups in MLN (**Figure 6C**). No significant changes were seen in the spleen at days 28 or 35 (**Figures 6D, E**), while they increased at day 50 with 7 and 14 days of HMO supplementation in the spleen (**Figure 6F**). The ratio of CD4+ to CD8+ T cells in MLN increased at day 35 with 14 days of HMO supplementation (**Figure 6H**).

**Figure 7:**
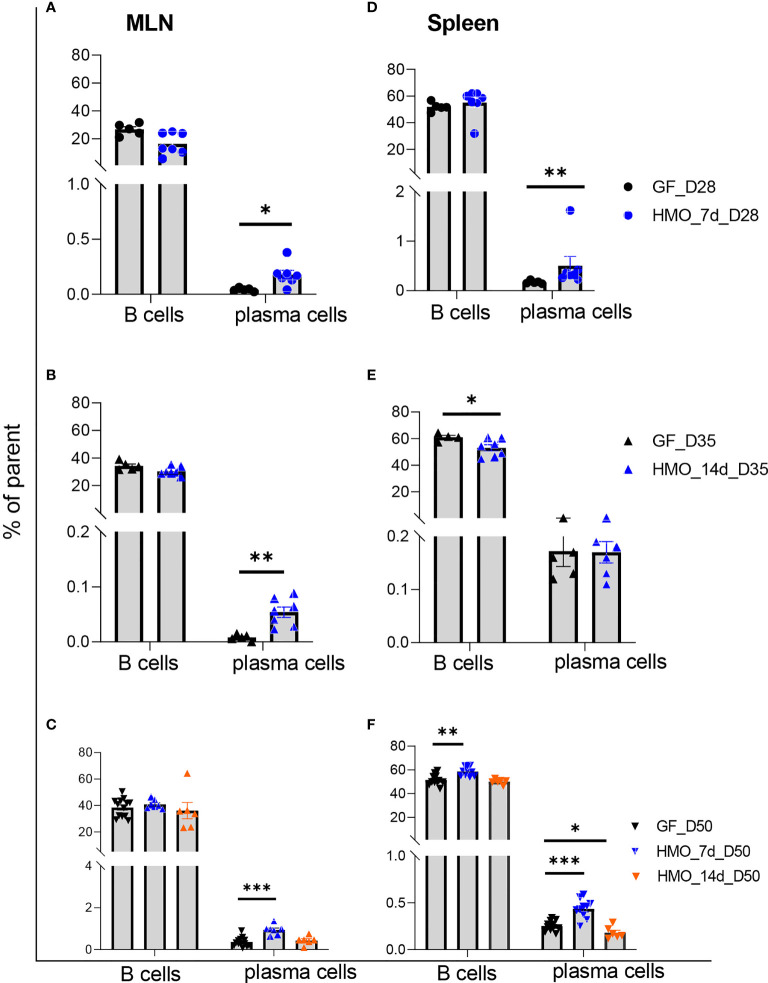
B cells (B220+) and plasma cells (B220- CD138+) in MLN and the spleen following HMO administration. **(A–C)** B cells and plasma cells in MLN. **(D–F)** B cells and plasma cells in the spleen. Data are shown as the percentage of the respective parent population (**Supplementary Figure 1B**). Each dot represents a mouse, and data are shown as mean ± SEM. Statistical analysis was performed using the Mann–Whitney test in GraphPad Prism Version 9.3.1 (www.graphpad.com). Statistical significance: *** p<0.001, **p<0.01, *p<0.05, ns, not significant. Groups: Control (GF_D28, GF_D35, and GF_D50) = GF mice euthanized either at 28 days of age (n= 5; M=2, F=3) or 35 days of age (n= 5; M=2, F=3) or 50 days of age (n= 16; M=8, F=8) respectively; HMO_7d_D28 (n= 7; M=3, F=4) = GF mice that received 100 μl of HMO through 7 consecutive days and euthanized at 28 days of age; HMO_14d_D35 (n= 7; M=3, F=4) = GF mice that received 100 μl of HMO through 14 days and euthanized at 35 days of age; HMO_7d_D50 (n= 11; M=6, F=5) = GF mice that received 100 μl of HMO through 7 consecutive days and euthanized at 50 days of age; HMO_14d_D50 (n= 6; M=5, F=1) = GF mice that received 100 μl of HMO through 14 consecutive days and euthanized at 50 days of age.

B220+ B-cell percentage in MLNs was not different between the GF and HMO-supplemented mice (**Figures 7A–C**). However, at days 28 and 35 and at day 50 in only the 7-day HMO group, plasma cells (B220- CD138+) were increased in MLNs. In the spleen, B cells were decreased in the 14-day HMO-supplemented group (**Figure 7E**). Interestingly, HMO supplementation increased B-cell percentage at day 50 in the spleen (**Figure 7F**). The plasma cell population was higher at day 28 as well as at day 50 with 7-day HMO supplementation (**Figures 7D, F**).

**Figure 8:**
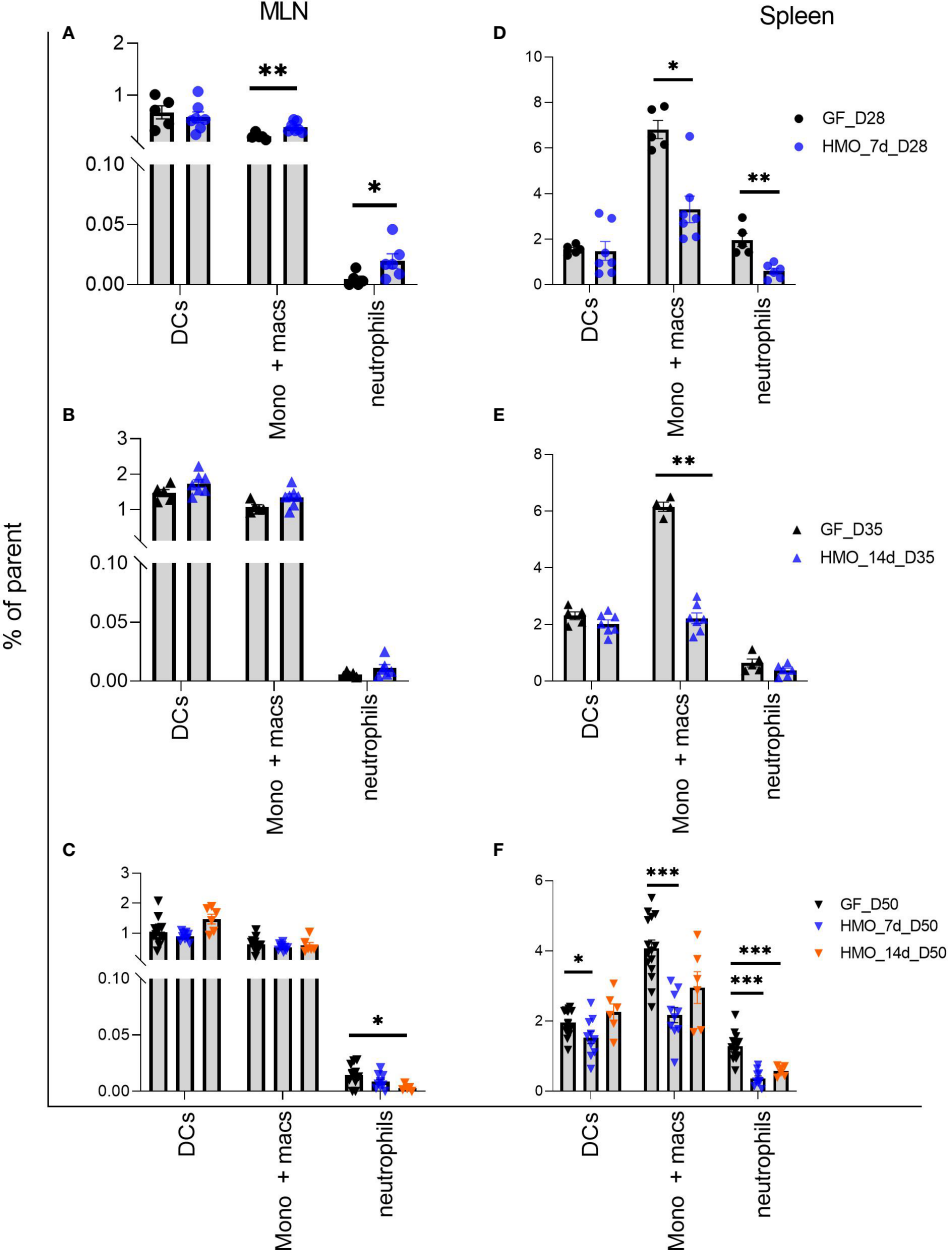
Neutrophils (PMNs) (B220- NK 1.1- CD11b+ Ly6G+), Monocytes/Macrophages (B220- NK 1.1- Ly6G- CD11b+) and dendritic cells (B220- NK 1.1- CD11c+ MHC-II+) in MLN and spleen following HMO administration. **(A–C)** Myeloid cell composition in MLN. **(D–F)** Myeloid cells composition in spleen. Data are shown as the percentage of respective parent population (**Supplementary Figure 1C**). Each dot represents a mouse and data are shown as mean ± SEM. Statistical analysis was performed using Mann–Whitney test in GraphPad Prism Version 9.3.1 (www.graphpad.com). Statistical significance: *** p<0.001, **p<0.01, *p<0.05, ns, not significant. Groups: Control (GF_D28, GF_D35, and GF_D50) = GF Mice euthanized either at 28 days of age (n= 5; M=2, F=3) or 35 days of age (n= 5; M=2, F=3) or 50 days of age (n= 16; M=8, F=8) respectively; HMO_7d_D28 (n= 7; M=3, F=4) = GF mice that received 100 ul of HMO through 7 consecutive days and euthanized at 28 days of age; HMO_14d_D35 (n= 7; M=3, F=4) = GF mice that received 100 ul of HMO through 14 days and euthanized at 35 days of age; HMO_7d_D50 (n= 11; M=6, F=5) = GF mice that received 100 ul of HMO through 7 consecutive days and euthanized at 50 days of age; HMO_14d_D50 (n= 6; M=5, F=1) = GF mice that received 100 ul of HMO through 14 consecutive days and euthanized at 50 days of age.

Among the three myeloid cell groups measured, there was no difference in dendritic cells (DCs) between control and HMO-supplemented groups in MLN (**Figures 8A–** **C**). Monocytes/macrophages were higher in MLN in a shorter duration of HMO supplementation (7 days) at day 28 (**Figure 8A**), while in the spleen, this population was lower in all HMO-supplemented groups (**Figures 8D–F**).

### 3.4 Humoral and Cell-Mediated Immune Responses

**Figure 9:**
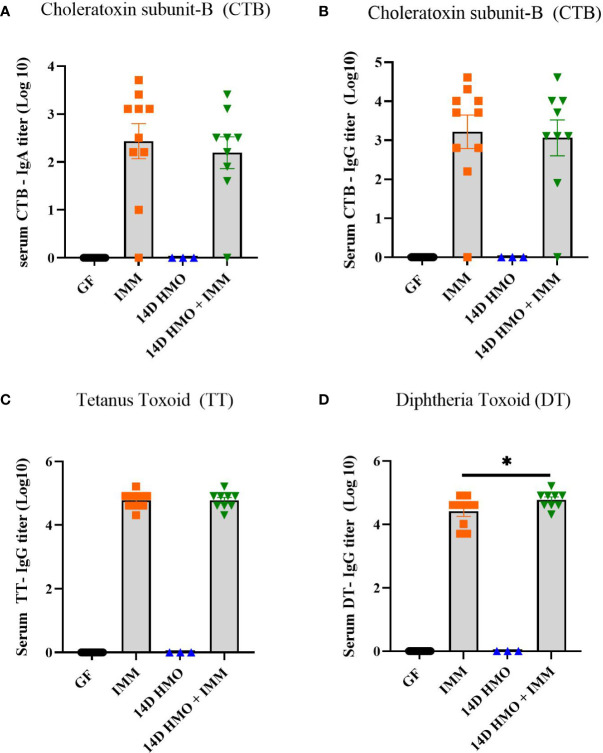
Serum IgA and IgG titers in immunized or HMO-supplemented groups. **(A)** CTB-specific serum IgA **(B)** CTB-specific IgG **(C)** TT-specific serum IgG **(D)** DT-specific IgG. GF control (n = 16, M = 8, F = 8), immunized (IMM) (n = 10, M = 6, F = 4), 14-day (14D) HMO (n = 3, F = 3) and 14D HMO + IMM (n = 9, M = 4, F = 5). Statistical significance between control and treatment groups were determined using one-way ANOVA with Bonferroni’s multiple comparison correction in GraphPad Prism Version 9.3.1 and adjusted p-value <0.05 was considered significant. Outliers were removed using ROUT (Q = 1%) in GraphPad Prism Version 9.3.1.* indicates significance P<0.05.

**Figure 10:**
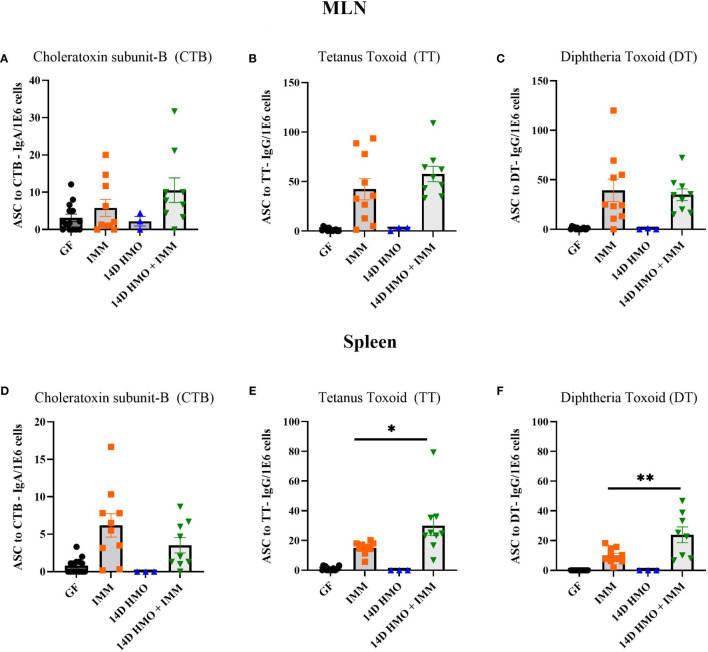
Immunization specific antibody-secreting cells (ASCs) from MLN and spleen, normalized per million cells. **(A–C)** CTB, TT and DT-specific ASCs from MLN. **(D–F)** CTB, TT and DT-specific ASCs from spleen. GF control (n = 16, M = 8, F = 8), immunized (IMM) (n = 10, M = 6, F = 4), 14D HMO (n = 3, F = 3) and 14D HMO + IMM (n = 9, M = 4, F = 5). Statistical significance between control and treatment groups were determined using one-way ANOVA with Bonferroni’s multiple comparisons correction in GraphPad Prism Version 9.3.1 and adjusted p-value <0.05 was considered significant. Outliers were removed using ROUT (Q = 1%) in GraphPad Prism Version 9.3.1. *P<0.05, **P<0.01.

**Figure 11:**
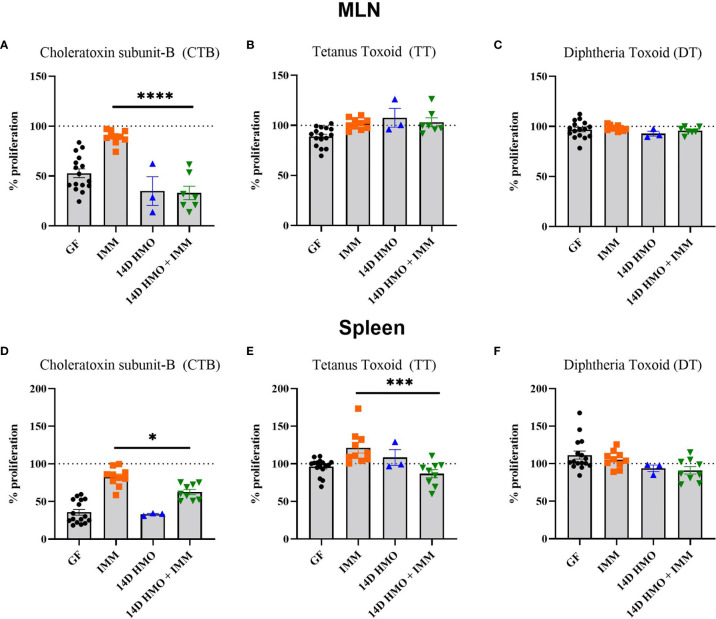
The proliferation response elicited from MLN or spleen cells to CTB, TT, or DT. **(A–C)** CT-, TT-, and DT-specific proliferation in MLN, respectively. **(D–F)** CT-, TT-, and DT-specific proliferation in spleen, respectively. Statistical significance between control and treatment groups were determined using one-way ANOVA with Bonferroni’s multiple comparison correction in GraphPad Prism Version 9.3.1 and the adjusted p- value <0.05 was considered significant. Outliers were removed using ROUT (Q = 1%) in GraphPad Prism Version 9.3.1. This indicates statistical significance.* P < 0.05, *** P < 0.005, ****P < 0.0001.

To assess the humoral immune response, we measured the antigen-specific IgA and IgG levels *via* ELISA in mice immunized with either choleratoxin or pediarix (immunization against diphtheria, tetanus, and pertussis), and there were no differences in the levels of CTB-IgA, CTB-IgG, and TT-IgG between GF-immunized and those supplemented with HMO for 14 days and immunized (**Figures 9A–C**). However, HMO supplementation increased the serum DT-IgG titer (**Figure 9D**) in comparison to GF-immunized mice. In addition, we assessed the mesenteric and systemic immune responses to CTB, TT, and DT by measuring a specific antibody response *ex vivo* in MLN and the spleen, respectively. The antibody-secreting cells (ASCs) of IgA to CTB and of IgG to TT/DT were quantified using ELISpot. In MLN, there was no difference between the GF-immunized and HMO-immunized groups for CTB, TT, or DT (**Figures 10A–C**). In the spleen, we found no difference in ASC for CTB between GF-immunized and HMO-immunized groups (**Figure 10D**). However, HMO supplementation showed increased ASC for TT and DT antigens compared to GF-immunized groups in the spleen (**Figures 10E, F**). Cell-mediated immune response was assessed by measuring cell proliferation in MLN and splenocytes after exposure to CTB, DT, and TT for 24 h. In both MLN and the spleen, proliferation was lower in HMO-immunized groups in comparison to GF-immunized ones (**Figures 11A, D**). In addition, in the spleen, HMO-immunized groups also had lower proliferation when comparted to GF-immunized mice (**Figure 11E**).

## 4 Discussion

Immediately after birth, newborns undergo a metabolic and physiological adaptation to extra-uterine life. Such alterations include the GIT microbiota colonization and establishment, immune system maturation, and intestinal development in response to the neonatal diet and exposure to dietary components (57–59). In addition, GIT maturation is a key element for physiologic differences in response to diet and pharmacokinetics (60) in infants relative to adults. Several prenatal and postnatal factors influence the neonates’ metabolic and immunological adaptations (61). However, the neonatal diet (human milk or milk formula) is a major contributor to these adaptations (62). For instance, human milk feeding stimulates the establishment of beneficial bacteria in the gut, as well as promotes the infants’ immune system maturation, likely by its composition (3, 63). Human milk contains different bioactive molecules, including microbiota, microRNAs, antibodies, anti-inflammatory peptides, hormones, and human milk oligosaccharides (HMOs), among others (64–66). Within the human milk composition, HMOs play an important role in improving the gut barrier function and enhancing the immune response in human milk–fed infants (67, 68). These HMO benefits have been attributed to the HMO–gut microbiota interaction by which HMOs serve as substrates to commensal bacteria, promote the growth of beneficial microbiota, and prevent pathogen binding to the epithelium (30, 69–72). However, the direct effects of HMO consumption in the absence of gut microbiota and the impact on gut and immune system remain to be determined. Thus, this study aimed to evaluate the HMOs’ effects in a GF mouse model.

The SI epithelium is composed of villi that contain enterocytes and goblet cells and of crypts that invaginate into the lamina propria containing stem cells and Paneth cells at its base (73, 74). In a murine model for necrotizing enterocolitis, HMOs pooled from human milk increased the expression of mucin 2 (*MUC2*), thus increasing mucus production, while a reduction in the intestinal permeability was detected by dextran permeability (75). In contrast to the histomorphometric findings in this current study, the daily administration of the HMO 2’FL from 2 to 16 days of age increased the intestinal villus heights and areas in suckling rats at day 8 (30), as well as higher villi in the distal SI tissue were reported in neonatal piglets fed with milk replacer supplemented with 2’FL (76). Higher villus height and crypt depth were observed in male C57BL/6 mice supplemented with 2’FL for 7 days compared to a control group at 56 days of age (77). However, other studies did not detect any difference in villus height in preterm pigs fed with a formula enriched with 2’FL for 5 days (78). Overall, the findings on intestinal gland depth and villus height reported in the present study might be associated with the lack of gut microbiota, which might play a role in absorption (79). In addition, the HMOs gavaged to mice in our study were a pool, while other studies were conducted with only 2’-FL, which is dominant in secretor mothers. It is possible that the influence of HMOs on the intestinal structure might be dependent on the composition of the HMOs and their interaction with the host microbiota. This further poses questions about whether single HMOs could be added to infant formulas in isolation, and whether that impacts gut and immune function has yet to be determined.

Regardless of time, HMO feeding upregulated the expression of several genes involved in transport, absorption, and secretion in the SI tissue. Most recently, the essential role of ST6GALNAC1 (ST6 N-acetylgalactosaminide alpha-2,6-sialyltransferase 6) in the sialylation of glycans on intestinal mucus showed gut homeostasis (80). In our study, a similar enzyme involved in sialylation (*St6galnac*) was altered by HMO supplementation in SI, suggesting a role of HMOs in upregulating the enzymes in mucus function and gut homeostasis. Therefore, these results seem to indicate that HMO feeding can, at least transiently, modulate the mucosal expression of developmentally regulated genes. Our findings align with previous reports that demonstrated that HMOs can resist enzymatic hydrolysis in the upper GIT, while intact HMOs might be absorbed in the SI (18, 81). The majority of HMOs consumed reach the LI, where they serve as substrates for bacterial metabolism (82) or are excreted intact (83, 84). However, to characterize the extent and nature of the gene expression modulated by HMO feeding in the absence of gut bacteria, we performed a set of the analyses of target genes through the combination of genes most strongly affected by both age and/or HMO feeding. These results highlight a striking impact of HMO feeding on host gene expression and unveil specific genes and pathways (e.g., mRNA metabolic processes) where the interaction between developmental expression programs and HMO feeding is prevalent.

At 28 and 35 days of age, specific pro-inflammatory cytokines had similar expression levels among the mice regardless of treatment. However, at 50 days of age, the 14 days of HMO feeding seemed to regulate the expression of *IL1B* and *IL1A* (e.g., cluster 5) compared to the control and 7 days of HMO-feeding group in the SI. These findings are in line with other *in vitro* approaches that reported the direct effects of HMOs in cytokine production (85). For instance, the HMO 6’-sialyllactose induced a reduction in the production of the tumor necrosis factor (TNF) in antigen-stimulated splenocytes, while an increase in the production of the anti-inflammatory cytokine interleukin 10 (IL-10) was detected in the MLNs of mice that received oral administration of 2’FL and 6’-sialyllactose (86). Persistent effects of HMO feeding were also detected at 50 days of age in the LI tissues of HMO-treated mice. HMO treatment upregulated the expression of immune response–related genes, including *FOXP3*, Ccl20, Cccr6, and receptor GPR68 (87, 88).

The expression of the chemokine *CCL20* was increased in the LI of HMO-treated mice at 50 days of age. This chemokine has antimicrobial properties as well as acts as a chemotactic factor for lymphocytes (89, 90). These findings are in line with *in vitro* approaches that reported a higher expression of *CCL20* in colonic epithelial cells treated with a pool of HMOs (91). Together, these findings suggest that an upregulation of specific immune response–related genes driven by HMOs can potentially enhance the protection against infections in infants by priming the neonate’s immune system.

Gut microbiota, along with the interaction with diet, influences the immune cell composition. HMOs can modulate the gut microbiota (e.g., *Bifidobacterium*) composition, which, in turn, impacts the immune response in the host, whereas microbiota, in turn, breaks down the HMOs (92, 93). In GF mice, however, the immune system development is impaired. We utilized this model to evaluate the HMOs’ role in the immune cell composition of MLN and the spleen in GF mice supplemented with HMOs. We observed that CD4+ T cells were higher at day 50 with 7 and 14 days of HMO supplementation in both MLN and spleen. Likewise, CD8+ T cells were also higher at day 50 with 7 and 14 days of HMO supplementation in the spleen. Although it did not pass the statistical threshold, a similar trend was seen in MLN as well (two-tailed p-values 0.061 and 0.053 at day 50 of 7 and 14 days of HMOs, respectively). Previous reports have shown that the length of breastfeeding impacts CD4 and CD8 cell numbers with no clear trend of CD4 or CD8; differences were observed based on the cohort and time of sample collection (94, 95), suggesting a knowledge gap. HMO supplementation did not change the B-cell population in MLN, whereas in the spleen, it was lower in the 14-day HMO group. B cells were not different between breastfed and formula-fed infants in a previous study (95). Interestingly, at day 50, B cells were higher in the 7-day HMO group. A novel observation of our study is that plasma cells were higher in all groups except in MLN at day 50 with 14-day HMO supplementation.

Among the myeloid cells, no difference was observed in CD11c+ MHC-II+ dendritic cells in MLN and it was decreased in the spleen at day 50 with 7-day HMO supplementation. HMOs can partially induce the semi-maturation of monocyte-derived dendritic cells *in vitro*, upregulating anti-inflammatory cytokines and the enhancement of regulatory T-cell development (34). Our findings suggest that in GF *in vivo* models, the HMOs might not impact dendritic cell composition, owing to the absence of microbiota. Another myeloid cell type, the neutrophil population, was increased due to 7-day HMO feeding at day 28 in MLN, while at day 50 in the 14-day HMO group, it was lower than the controls. Monocytes/macrophages were higher in MLN with 7-day HMO supplementation, while in the spleen, this population was lower in all HMO-supplemented groups. It is also interesting that neutrophils and monocytes/macrophages showed opposite trends in MLN and spleen with a shorter duration of HMO supplementation (7 days of HMOs at day 28). These findings suggest that even in the absence of microbiota, HMOs can induce some changes in lymphocyte subpopulation distribution.

Breastfed versus formula-fed infants have shown to have a robust immune response to vaccines (96). In addition, recently, 2’-FL supplementation showed higher vaccine response in a murine influenza vaccination model (38). In our study, DT and TT antibody-secreting cells were higher in spleen and the TT-IgG antibody response was also higher in the HMO-supplemented group in comparison to controls. Finally, the effects can be influenced by the lymphoid organs, duration of HMO supplementation, and age of the mice. The results observed in our study were only in the presence of HMOs, while human milk has several bioactives and microbiota interaction likely impacts the local and systemic immune cell responses. All these data suggest that HMOs have a direct effect on the gut and immune response. Future research will have to determine the functional relevance of these differential cell population changes in response to HMOs and in the absence or presence of microbiota. Furthermore, the role of additional bioactives (i.e., miRNAs and IgA) of human milk in driving immune cell composition and function and vaccine response will be important to understand to fill the gap in knowledge.

## 5 Conclusions

The influence of HMOs on gut microbiota composition and immunity has been shown in breastfed infants. Such positive health outcomes have been attributed to the mixture of oligosaccharides found in human milk. In the present study, we evaluated the oral administration of pooled HMOs isolated from human milk for either 7 or 14 days in GF mice; this design might not represent the typical exposure of breastfeeding with human milk matrix in infants. Therefore, the results from this study should not be directly extrapolated. We report that HMOs exert some direct effects in the absence of host microbiota, including the regulation of genes involved in cellular and inflammatory pathways. HMO administration decreased the intestinal villus and crypt, as well as distal gut gland depth, and altered immune cell response. Overall, HMO administration appears to promote immunoregulatory effects at the gene expression level in the absence of gut microbiota. Finally, HMO interactions with the host microbiota might be needed for optimal intestinal adaptation.

## Data Availability Statement

The datasets for this study can be found in the Bio Project ID PRJNA814680 [http://www.ncbi.nlm.nih.gov/bioproject/814680].

## Ethics Statement

The animal study was reviewed and approved by University of Arkansas for Medical Sciences.

## Author Contributions

LY conceptualized the study. AS and DC conducted RNA-seq data analyses and statistical analysis. MG conducted flow cytometry data analyses. LB provided the HMO used as treatment and contributed to data interpretation; KM and PT conducted the mice experiments. CS provided veterinary support. AE, KM, PT, and FR performed sample collection. CR performed sample sequencing. TH contributed to the editing of the manuscript. KW conducted a statistical analysis of histomorphometric data. TL conducted the histomorphometric analysis. FR and LY contributed to data interpretation and wrote the manuscript. FR and LY have the primary responsibility for the manuscript. All the authors revised the manuscript draft and agreed to the published version.

## Funding

This work was supported by NIGMS [P20GM121293] and partly by USDA-ARS [6026-51000-010-06S].

## Conflict of Interest

The authors declare that the research was conducted in the absence of any commercial or financial relationships that could be construed as a potential conflict of interest.

## Publisher’s Note

All claims expressed in this article are solely those of the authors and do not necessarily represent those of their affiliated organizations, or those of the publisher, the editors and the reviewers. Any product that may be evaluated in this article, or claim that may be made by its manufacturer, is not guaranteed or endorsed by the publisher.

## Supplementary Material

Supplementary Figure 1 Representative gating plots for T, B and myeloid cells. A. Representative gating for T cells (CD3+CD4+ and CD4-CD3+CD8+) B. Representative gating for B cells (B220+) & plasma cells (B220-CD138+) C. Representative gating for myeloid cells Neutrophils (B220- NK1.1- CD11b+ Ly6G+), Monocytes/macrophages (Mono + macs) (B220- NK1.1- Ly6G- CD11b+) and dendritic cells (B220- NK1.1- CD11c+ MHCII+)

Supplementary Figure 2 Violin plots of variance partition analysis for all experimental factors analyzed in **(A)** small intestine (SI) and **(B)** large intestine (LI) samples.

Supplementary Figure 3 Expression profile of gene clusters obtained from model-based analysis of highly variable genes in small intestine samples. For each condition, shown are barplots representative of at least five biological replicates. The number of genes classified in each cluster is displayed on top.

Supplementary Figure 4 Expression profile of gene clusters obtained from model-based analysis of highly variable genes in LI samples. For each condition, shown are barplots representative of at least five biological replicates. The number of genes classified in each cluster is displayed on top.

Supplementary Data Sheet 1 Per sample estimated proportions of different cell types obtained by de-convolution of bulk RNA-Seq data using single-cell expression signatures from the Tabula Muris reference dataset.

Supplementary Data Sheet 2 Gene-wise expression variance partition using a global (including both small and large intestine samples) multivariate model.

Supplementary Data Sheet 3 Pathway enrichment results for genes most associated with sample location (small vs. large intestine).

Supplementary Data Sheet 4 Gene-wise expression variance partition for location-specific (separately for small and large intestine samples) multivariate models.

Supplementary Data Sheet 5 Pathway enrichment results for highly variable genes (genes most associated with both Age and HMO feeding) in small intestine samples.

Supplementary Data Sheet 6 Pathway enrichment results for highly variable genes (genes most associated with both age and HMO feeding) in large intestine samples.

Supplementary Table 1 The Panel of antibodies used for staining mesenteric lymph node and spleen cells.

Supplementary Table 2 Analysis of Deviance Table (Type II Tests) for group^1^, sex, and their interactions at day 28 and 35. ^1^Groups: Control = germ-free mice euthanized either at 28 days of age or 35 days of age; HMO 7 d = germ-free mice that received 100 µl of HMO (15 mg/day) through 7 consecutive days and euthanized at 28 days of age; HMO 14 d = germ-free mice that received 100 µl of HMO (15 mg/day) through 14 consecutive days and euthanized at 35 days of age.

Supplementary Table 3 Permanova for group^1^, sex, and their interactions at day 50.^1^Groups: Control = germ-free mice euthanized at 50 days of age; HMO 7 d = germ-free mice that received 100 µl of HMO (15 mg/day) through 7 consecutive days and euthanized at 50 days of age; HMO 14 d = germ-free mice that received 100 µl of HMO (15 mg/day) through 14 consecutive days and euthanized at 50 days of age.
