# Supplementary material for: Human Milk Oligosaccharides Impact Cellular and Inflammatory Gene Expression and Immune Response
Source: Front Immunol. 2022 Jun 29;13:907529. doi: 10.3389/fimmu.2022.907529 (PMC9278088; doi:10.3389/fimmu.2022.907529)
Supplement: Supplementary Figure 1 — Representative gating plots for T, B and myeloid cells. A. Representative gating for T cells (CD3+CD4+ and CD4-CD3+CD8+) B. Representative gating for B cells (B220+) & plasma cells (B220-CD138+) C. Representative gating for myeloid cells Neutrophils (B220- NK1.1- CD11b+ Ly6G+), Monocytes/macrophages (Mono + macs) (B220- NK1.1- Ly6G- CD11b+) and dendritic cells (B220- NK1.1- CD11c+ MHCII+) [file Image_1.pdf]

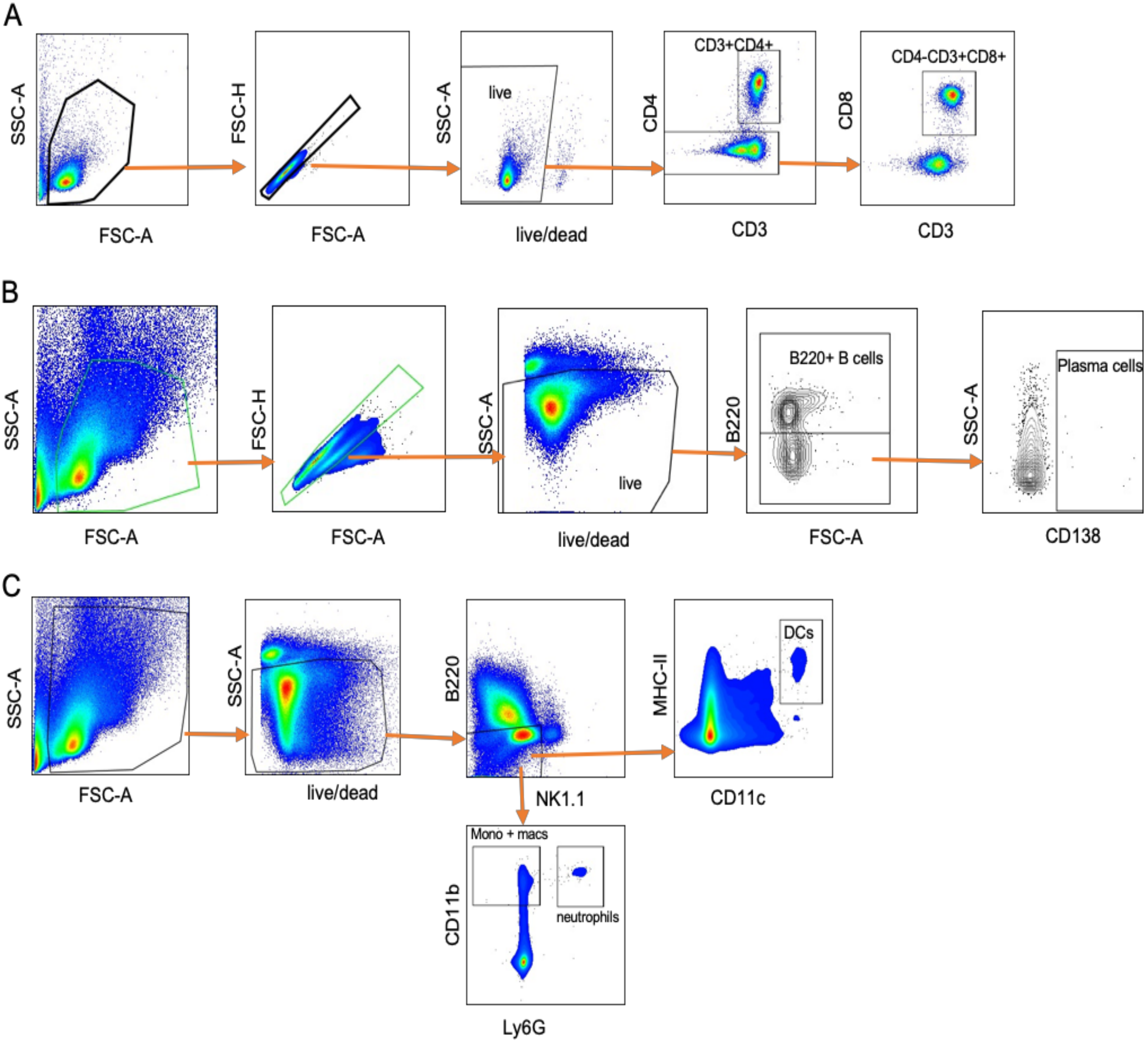

Supplementary figure 1: Representative gating shown for T, B and myeloid cells.
A. Representative gating for T cells (CD3+CD4+ and CD4-CD3+CD8+) B. Representative gating for B cells (B220+) & plasma cells (B220-CD138+) C. Representative gating for myeloid cells Neutrophils (B220- NK1.1- CD11b+ Ly6G+), Monocytes/macrophages (Mono + macs) (B220- NK1.1- Ly6G- CD11b+) and dendritic cells (B220- NK1.1- CD11c+ MHCII+)
