# Supplementary figures and images for: Human Milk Oligosaccharides Impact Cellular and Inflammatory Gene Expression and Immune Response
Source: Front Immunol. 2022 Jun 29;13:907529. doi: 10.3389/fimmu.2022.907529 (PMC9278088; doi:10.3389/fimmu.2022.907529)

A

## Small Intestine

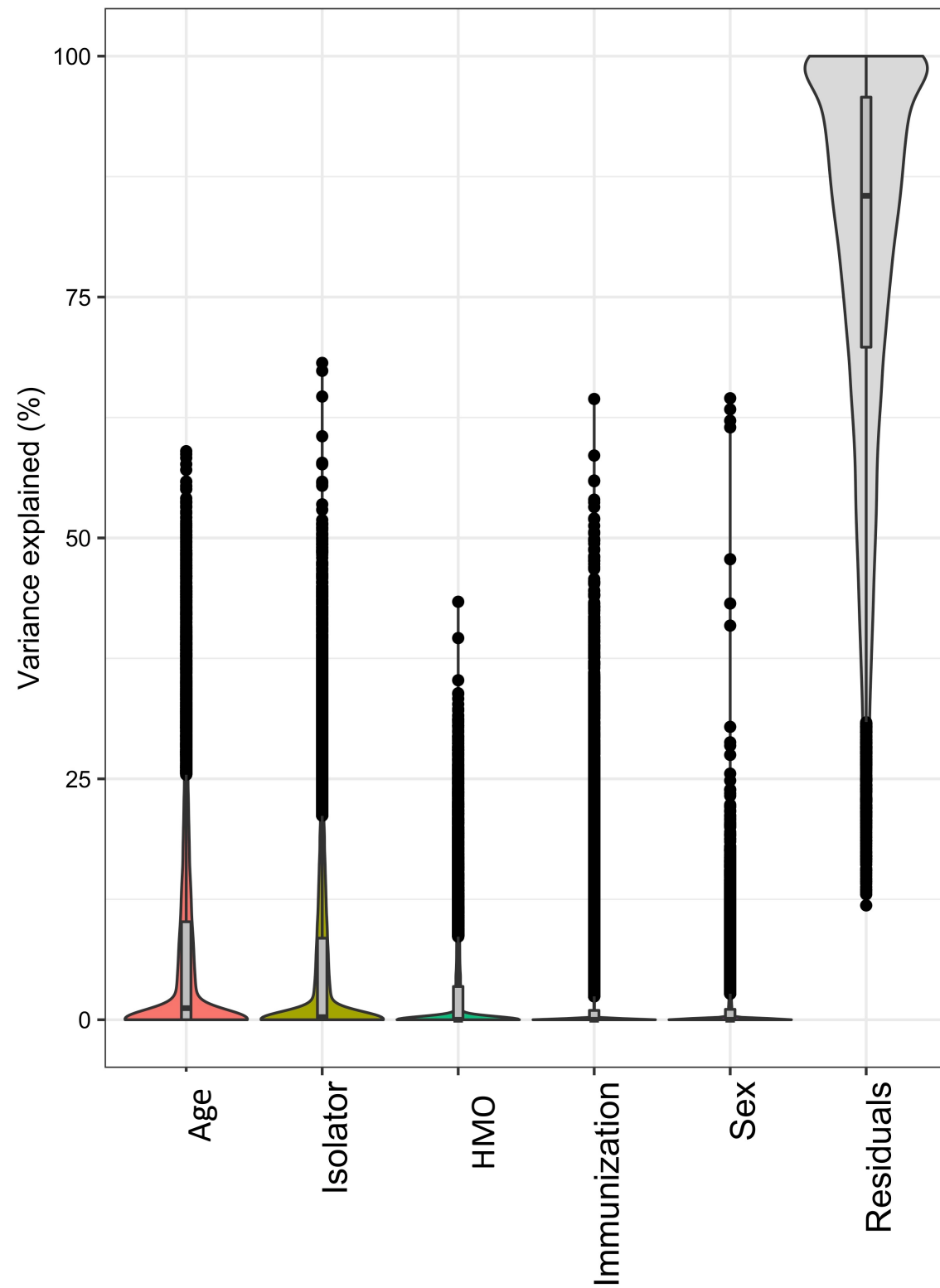

B

## Large Intestine

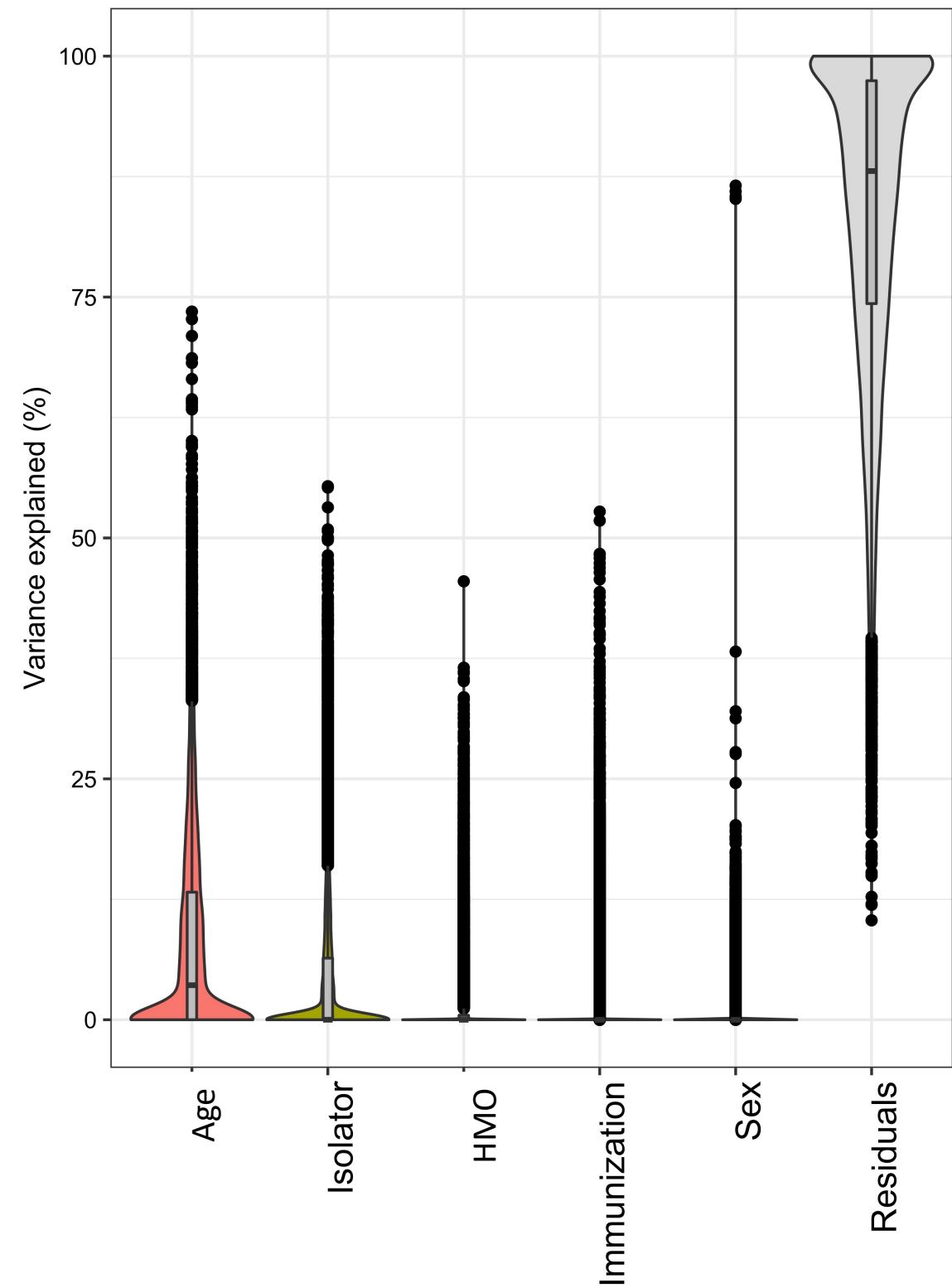

Supplement: Supplementary Figure 2 — Violin plots of variance partition analysis for all experimental factors analyzed in (A) small intestine (SI) and (B) large intestine (LI) samples. [file Image_2.pdf]

Mean expression (VSD)

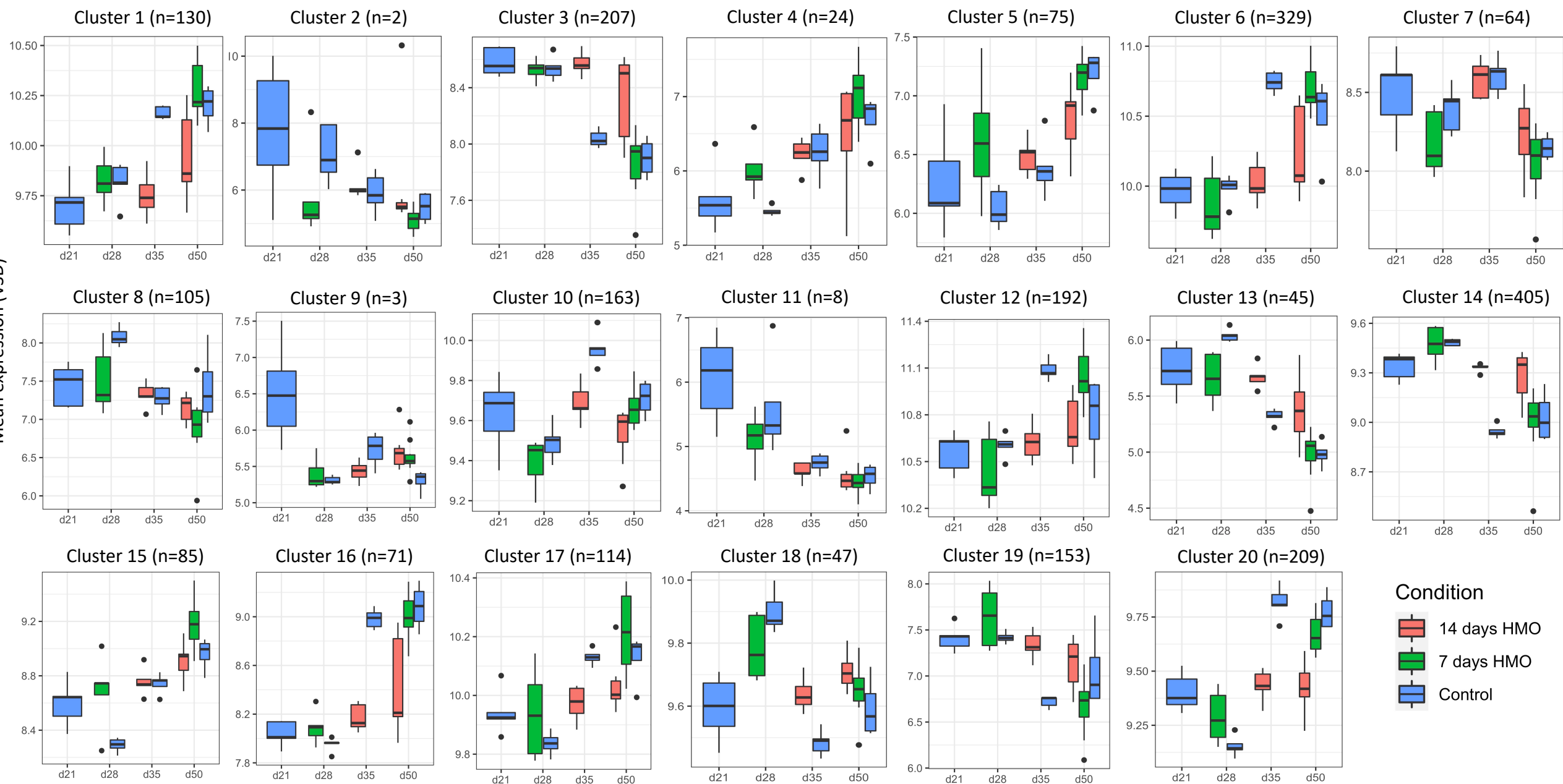

Supplement: Supplementary Figure 3 — Expression profile of gene clusters obtained from model-based analysis of highly variable genes in small intestine samples. For each condition, shown are barplots representative of at least five biological replicates. The number of genes classified in each cluster is displayed on top. [file Image_3.pdf]

Mean expression (VSD)

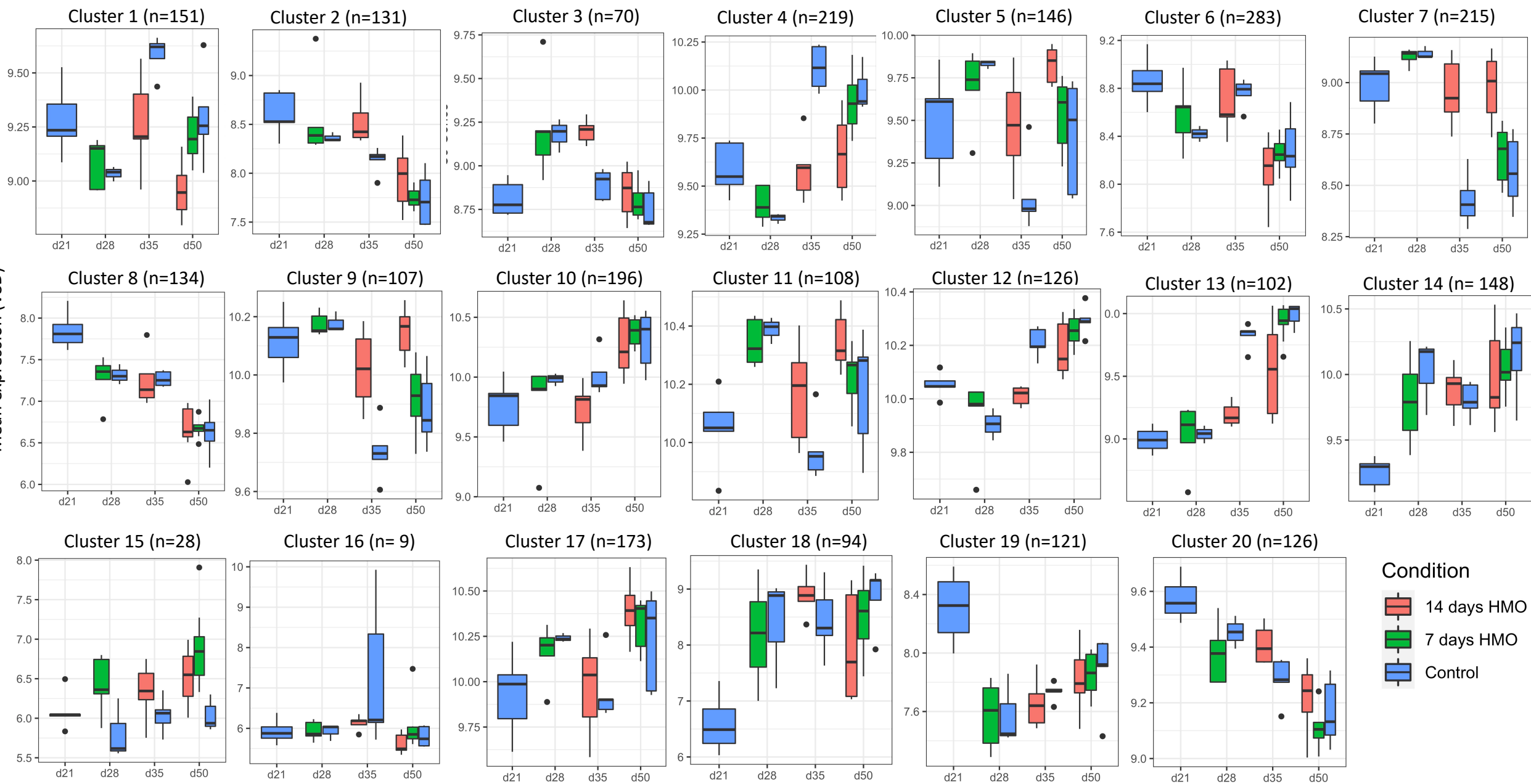

Supplement: Supplementary Figure 4 — Expression profile of gene clusters obtained from model-based analysis of highly variable genes in LI samples. For each condition, shown are barplots representative of at least five biological replicates. The number of genes classified in each cluster is displayed on top. [file Image_4.pdf]
