# Supplementary material for: Human Milk Oligosaccharides Impact Cellular and Inflammatory Gene Expression and Immune Response
Source: Front Immunol. 2022 Jun 29;13:907529. doi: 10.3389/fimmu.2022.907529 (PMC9278088; doi:10.3389/fimmu.2022.907529)
Supplement: Supplementary Table 1 — The Panel of antibodies used for staining mesenteric lymph node and spleen cells. [file Table_1.docx]

**Supplementary Table 1**: The Panel of antibodies used for staining mesenteric lymph node and spleen cells.

| **Marker** | **Type** | **Company** | **Catalog #** |
| --- | --- | --- | --- |
| Zombie Aqua fixable | Surface | Bio-legend | 423101 |
| CD3- APC | Surface | Bio-legend | 100236 |
| CD4 – APC Cy7 | Surface | Bio-legend | 100414 |
| CD8 – PerCP/Cy5.5 | Surface | Bio-legend | 140418 |
| B220 –APC Cy7 | Surface | Bio-legend | 103224 |
| CD138- PE Cy7 | Surface | Bio-legend | 142514 |
| Ly6G-APC | Surface | Bio-legend | 127613 |
| CD11b-PE/Dazzle594 | Surface | Bio-legend | 101256 |
| CD11c-PE | Surface | Bio-legend | 117308 |
| NK1.1-AF488 | Surface | Bio-legend | 108718 |
| MHCII – AF700 | Surface | Bio-legend | 107622 |

**Supplementary Table 2**. Analysis of Deviance Table (Type II Tests) for group^1^, sex, and their interactions at day 28 and 35.

| **Small intestinal crypt** | **Chisq model** | ***P*r (>Chisq)** |
| --- | --- | --- |
| Group | 26.89 | ˂ 0.001 |
| Sex | 6.43 | 0.01 |
| Group:Sex | 13.74 | ˂ 0.01 |
|  |  |  |
| **Small intestinal villi** | **Chisq model** | ***P*r (>Chisq)** |
| Group | 29.04 | ˂ 0.001 |
| Sex | 5.61 | 0.02 |
| Group:Sex | 6.20 | 0.10 |
|  |  |  |
| **Large intestine** | **Chisq model** | ***P*r (>Chisq)** |
| Group | 7.88 | 0.05 |
| Sex | 5.65 | 0.02 |
| Group:Sex | 7.53 | 0.06 |
|  |  |  |
| **Cecum** | **Chisq model** | ***P*r (>Chisq)** |
| Group | 14.05 | ˂ 0.01 |
| Sex | 0.90 | 0.34 |
| Group:Sex | 6.37 | 0.10 |

^1^Groups: Control = germ-free mice euthanized either at 28 days of age or 35 days of age; HMO 7 d = germ-free mice that received 100 µL of HMO (15mg/day) through 7 consecutive days and euthanized at 28 days of age; HMO 14 d = germ-free mice that received 100 µL of HMO (15mg/day) through 14 consecutive days and euthanized at 35 days of age.

**Supplementary Table 3**. Permanova for group^1^, sex, and their interactions at day 50.

| **Small intestinal crypt** | **F-value** | ***P*-value** |
| --- | --- | --- |
| Group | 10.84 | ˂ 0.001 |
| Sex | 0.16 | 0.68 |
| Group:Sex | 3.60 | 0.03 |
|  |  |  |
| **Small intestinal villi** | **F-value** | ***P*-value** |
| Group | 28.02 | ˂ 0.001 |
| Sex | 0.25 | 0.62 |
| Group:Sex | 2.40 | 0.09 |
|  |  |  |
| **Large intestine** | **F-value** | ***P*-value** |
| Group | 15.65 | ˂ 0.001 |
| Sex | 9.83 | 0.002 |
| Group:Sex | 2.07 | 0.13 |
|  |  |  |
| **Cecum** | **F-value** | ***P*-value** |
| Group | 10.10 | ˂ 0.001 |
| Sex | 26.60 | ˂ 0.001 |
| Group:Sex | 1.45 | 0.23 |

^1^Groups: Control = germ-free mice euthanized at 50 days of age; HMO 7 d = germ-free mice that received 100 µL of HMO (15mg/day) through 7 consecutive days and euthanized at 50 days of age; HMO 14 d = germ-free mice that received 100 µL of HMO (15mg/day) through 14 consecutive days and euthanized at 50 days of age.
